# Protein expression and gene editing in monocots using foxtail mosaic virus vectors

**DOI:** 10.1002/pld3.181

**Published:** 2019-11-22

**Authors:** Yu Mei, Bliss M. Beernink, Evan E. Ellison, Eva Konečná, Anjanasree K. Neelakandan, Daniel F. Voytas, Steven A. Whitham

**Affiliations:** ^1^ Department of Plant Pathology and Microbiology Iowa State University Ames IA USA; ^2^ Department of Genetics, Cell Biology and Development Center for Genome Engineering Center for Precision Plant Genomics University of Minnesota St. Paul MN USA; ^3^ Department of Genetics, Development and Cell Biology Iowa State University Ames IA USA

**Keywords:** Cas9, CRISPR, gene editing, gene expression, guide RNA, virus

## Abstract

Plant viruses can be engineered to carry sequences that direct silencing of target host genes, expression of heterologous proteins, or editing of host genes. A set of foxtail mosaic virus (FoMV) vectors was developed that can be used for transient gene expression and single guide RNA delivery for Cas9‐mediated gene editing in maize, *Setaria viridis*, and *Nicotiana benthamiana*. This was accomplished by duplicating the FoMV capsid protein subgenomic promoter, abolishing the unnecessary open reading frame 5A, and inserting a cloning site containing unique restriction endonuclease cleavage sites immediately after the duplicated promoter. The modified FoMV vectors transiently expressed green fluorescent protein (GFP) and bialaphos resistance (BAR) protein in leaves of systemically infected maize seedlings. GFP was detected in epidermal and mesophyll cells by epifluorescence microscopy, and expression was confirmed by Western blot analyses. Plants infected with FoMV carrying the *bar* gene were temporarily protected from a glufosinate herbicide, and expression was confirmed using a rapid antibody‐based BAR strip test. Expression of these proteins was stabilized by nucleotide substitutions in the sequence of the duplicated promoter region. Single guide RNAs expressed from the duplicated promoter mediated edits in the *N. benthamiana Phytoene desaturase* gene, the *S. viridis Carbonic anhydrase 2* gene, and the maize *HKT1* gene encoding a potassium transporter. The efficiency of editing was enhanced in the presence of synergistic viruses and a viral silencing suppressor. This work expands the utility of FoMV for virus‐induced gene silencing (VIGS), virus‐mediated overexpression (VOX), and virus‐enabled gene editing (VEdGE) in monocots.

## 1 INTRODUCTION

Plant viruses provide surprisingly versatile technology platforms enabling the expression of a wide array of coding and non‐coding sequences in plants (Pasin, Menzel, & Daros, 2019). Viruses engineered to carry heterologous open reading frames (ORFs) can express the encoded proteins (VOX). Viruses that carry fragments of plant genes in sense and antisense orientation cause virus‐induced gene silencing (VIGS) of the targeted sequence, or microRNA inserts can initiate silencing with high specificity. Most recently, it has been demonstrated that plant viruses or their derivatives can be used to deliver CRISPR‐Cas reagents consisting of single guide RNAs (gRNAs), DNA repair templates, and site‐specific nucleases such as Cas9 (Ali et al., 2015; Ali, Eid, Ali, & Mahfouz, 2018; Butler, Baltes, Voytas, & Douches, 2016; Cody, Scholthof, & Mirkov, 2017; Dahan‐Meir et al., 2018; Gao et al., 2019; Gil‐Humanes et al., 2017; Jiang et al., 2019; Mahas, Ali, Tashkandi, & Mahfouz, 2019; Wang et al., 2017). These capabilities show that plant viruses can be useful biotechnological tools for gene function studies in plants, and they can have practical applications as well (Pasin et al., 2019; Zaidi & Mansoor, 2017).

The development of viral vectors for monocot plants has been emerging rapidly in recent years (Lee, Hammond‐Kosack, & Kanyuka, 2015). To date, at least eight different viruses have been developed into viral vectors for monocots, including barley stripe mosaic virus (BSMV) (Lee, Hammond‐Kosack, & Kanyuka, 2012; Scofield, Huang, Brandt, & Gill, 2005), brome mosaic virus (BMV) (Ding, Schneider, Chaluvadi, Mian, & Nelson, 2006), cymbidium mosaic virus (CymMV) (Hsieh et al., 2013), rice tungro bacilliform virus (RTBV) (Purkayastha, Mathur, Verma, Sharma, & Dasgupta, 2010), wheat streak mosaic virus (WSMV) (Choi, Stenger, Morris, & French, 2000; Tatineni, McMechan, Hein, & French, 2011), bamboo mosaic virus (BaMV) together with its associated satellite RNA (Liou, Huang, Hu, Lin, & Hsu, 2014), cucumber mosaic virus (CMV) (Wang et al., 2016), barley yellow striate mosaic virus (BYSMV) (Gao et al., 2019), and foxtail mosaic virus (FoMV) (Bouton et al., 2018; Liu et al., 2016; Mei & Whitham, 2018; Mei, Zhang, Kernodle, Hill, & Whitham, 2016). Seven of the virus vectors are designed for VIGS applications (BSMV, BMV, CymMV, RTBV, BaMV, CMV, and FoMV), and four can be used for systemic gene expression (BSMV, BYSMV, FoMV, and WSMV). BYSMV was also shown to deliver both Cas9 and a single guide RNA to *N. benthamiana* where it induced site‐specific gene edits at the infiltrated site (Gao et al., 2019).

Previous work by us and others demonstrated that FoMV is capable of systemic infection and inducing VIGS in maize (Liu et al., 2016; Mei & Whitham, 2018; Mei et al., 2016) or expressing proteins (Bouton et al., 2018). FoMV is a member of the genus *Potexvirus*, which has a single‐stranded, positive‐sense genomic RNA. The genome structure of FoMV is similar to potato virus X (PVX), which is the type member of the potexviruses. These viruses typically contain five major open reading frames (ORF) (Bruun‐Rasmussen, Madsen, Johansen, & Albrechtsen, 2008; Huisman, Linthorst, Bol, & Cornelissen, 1988; Robertson, French, & Morris, 2000). ORF1 encodes the RNA‐dependent RNA polymerase (RdRp), which is necessary for viral RNA replication and subgenomic messenger RNA (sgRNA) synthesis (Draghici & Varrelmann, 2009). The overlapping ORFs 2, 3, and 4 are known as the triple gene block (TGB) with functions in virus movement and suppression of host defense (Verchot‐Lubicz, 2005). ORF5 encodes the coat protein (CP), which is indispensable for virus assembly and cell‐to‐cell movement (Cruz, Roberts, Prior, Chapman, & Oparka, 1998). In addition to the five ORFs, the FoMV genome has a unique ORF5A that initiates 144 nucleotides upstream of the CP, but it is not required for replication or for systemic infection of *N. benthamiana* or barley (Robertson et al., 2000).

Recently, the FoMV‐based viral vectors have been developed for both VIGS and gene expression (Bouton et al., 2018; Liu et al., 2016; Liu & Kearney, 2010; Mei et al., 2016). Two versions of FoMV‐based VIGS vectors and their applications in maize and other monocots have been reported. Our original FoMV‐VIGS vector carried the insertion site for target gene fragments immediately after the stop codon of ORF5, which worked well for VIGS, but it cannot be used for gene expression (Mei et al., 2016). The FoMV‐VIGS vector reported by Liu et al. (2016) is designed to carry target sequences for silencing under the control of a duplicated FoMV CP subgenomic promoter. Inverted‐repeats carried at this position were most efficient at inducing VIGS (Liu et al., 2016). Bouton et al. (2018) also duplicated the CP promoter and demonstrated that FoMV could be used to transiently express marker and fungal effector proteins from this position in *N. benthamiana*, wheat, and maize. Disarmed FoMV vectors for transient gene expression have also been developed by substitution of the TGB or CP with genes of interest. In the presence of a viral RNA silencing suppressor, this set of vectors achieves high protein expression at the site of inoculation, but the recombinant virus cannot spread systemically (Liu & Kearney, 2010).

Here, we further investigated strategies to systemically express proteins in maize from FoMV using a duplicated CP strategy similar to Bouton et al. (2018). Protein expression was demonstrated using the genes encoding bialaphos resistance (*bar*) and green fluorescent protein (*GFP*), and time courses over plant development were used to understand insert stability and limitations of the vector for protein expression. We mapped subgenomic mRNA transcription start sites and modified duplicated CP promoter sequences to explore avenues for better understanding FoMV gene expression and increasing stability of heterologous sequences. To demonstrate applications of FoMV in editing plant genes, we expressed single gRNAs from the duplicated CP promoter sequence in *N. benthamiana*, *Setaria viridis*, and maize plants carrying Cas9 transgenes. Systemic gene editing was observed in leaves of all three species, and it was enhanced in the presence of an RNA silencing suppressor or a synergistic virus, demonstrating that FoMV can enable gene editing through the expression of functional gRNAs.

## 2 MATERIALS AND METHODS

### 2.1 Plant material and virus inoculation

The parent pFoMV‐V infectious clone from which all FoMV clones were derived was previously described (Mei et al., 2016). The SCMV virus isolate was originally named MDMV‐B and designated Iowa 66‐188 [ATCC‐PV53]) (Ford, Bucholtz, & Lambe, 1967; Hill, Ford, & Benner, 1973). The viruses were propagated in sweet corn (*Zea mays* L. ‘Golden x Bantam’; American Meadows). Virus‐infected leaf sap was prepared by grinding infected leaves in 50 mM potassium phosphate buffer, pH 7.0. Sweet corn plants at the two‐leaf stage were mechanically inoculated by rubbing leaf sap on new leaves dusted with 600‐mesh Carborundum. To inoculate plants with pFoMV infectious DNA clones, leaves of one‐week‐old seedlings were inoculated by particle bombardment using a Biolistic PDS‐1000/He system (Bio‐Rad Laboratories), 1.0‐µm gold particles coated with 1 µg of pFoMV DNA, and 1,100‐psi rupture disks at a distance of 6 cm as previously described (Mei & Whitham, 2018). Plants were placed in the dark for 12 hr before and after inoculation and then maintained in a greenhouse room with a thermostat set to 20–22°C with a 16‐hr photoperiod. *N. benthamiana* plants were grown in a growth room at 22°C with a 16‐hr photoperiod, and the *N. benthamiana* Cas9 line was previously described (Baltes et al., 2015). The *S. viridis* Cas9 line was generated by transforming strain A10 with a wheat codon‐optimized Cas9 gene expressed from the maize ubiquitin promoter (*ZmUbi)* (Cermak et al., 2017; Van Eck, Swartwood, Pidgeon, & Maxson‐Stein, 2017). The maize Cas9 line was described by Char et al. (2017).

### 2.2 Construction of FoMV‐derived vectors

A three‐step strategy was used to make pFoMV‐V‐derived expression vectors. All oligonucleotide primers are listed in Table S1. In step 1, pFoMV‐V was modified to disrupt the predicted start codon of ORF5A. In PCR reaction A, primer pairs 5AmuS1 and 5AmuA1 were used to amplify a product from pFoMV‐V and the product was gel extracted. In PCR reaction B, primer pair 5AmuS2 and 5AmuA2 was used with pFoMV‐V as template and the product was gel extracted. In overlapping PCR reaction C, primer pair 5AmuS1 and 5AmuA2 was used with PCR products A and B as templates. PCR product C was digested with restriction enzymes *Sac*II and *Sal*I, gel extracted, and ligated into pFoMV‐V that had been digested with the same enzymes to produce the pFoMV‐V‐Δ5A.

In step 2, a multiple cloning site was added into pFoMV‐V‐Δ5A. In PCR reaction D, primer pairs 5AmuS1 and 201DPA1 were used to amplify a product from pFoMV‐V‐Δ5A and the product was gel extracted. In PCR reaction E, primer pairs 201DPS1 and 5AmuA2 were used with pFoMV‐V‐Δ5A as template and the product was gel extracted. In overlap PCR reaction F, primer pair 5AmuS1 and 5AmuA2 was used with PCR products D and E as templates. PCR product F was digested with restriction enzymes *Sac*II and *Sal*I, gel extracted, and ligated into pFoMV‐V that had been digested with the same enzymes to produce the pFoMV‐V‐Δ5A‐MSC, which contains *Pml*I, *Bsu36*I, *Hpa*I and *Psp*OMI.

In step 3, the duplicated CP subgenomic promoters were added to pFoMV‐V‐Δ5A‐MSC. Oligonucleotides DPS and DPA were synthesized and annealed to form double‐strand DNA fragment that contained the putative subgenomic promoter for CP (nucleotides 5280–5333) and *Bsu36*I recognition site at 3’ end. The annealed product was digested with *Bsu36*I and ligated into pFoMV‐V‐Δ5A‐MSC that had been digested with *Pml*I and *Bsu36*I to produce the pFoMV‐DP. The pFoMV‐DC was constructed similarly using synthesized oligonucleotides DCS and DCA.

### 2.3 Generation of FoMV constructs for protein and RNA expression

*Bar* was amplified by PCR using pBPMV‐IA‐GFP‐BAR (Zhang, Bradshaw, Whitham, & Hill, 2010) as a template with primer pair BAR Bsu36I and BAR HpaI. The product was cloned into pGEM‐T Easy (Promega) and sequenced for verification. The *bar* gene was released by *Bsu*36I and *Psp*OMI double digestion and ligated into similarly digested pFoMV‐DP or pFoMV‐DC to generate the construct pFoMV‐DP‐BAR or pFoMV‐DC‐BAR. *GFP* was amplified by PCR using pSITE 2CA (Chakrabarty et al., 2007) as a template with primer pairs GFP Bsu36I and GFP PspOMI. pGFP was generated by cloning the product into pGEM‐T Easy, and it was verified by sequencing. pGFP was digested with *Bsu*36I and *Psp*OMI and ligated into similarly digested pFoMV‐DC to generate pFoMV‐DC‐GFP.

### 2.4 RT‐PCR analysis

Non‐infected wild‐type leaves or leaves of plants infected by pFoMV‐V, pFoMV‐V‐Δ5A, pFoMV‐DP, pFoMV‐DC, pFoMV‐DP‐BAR, pFoMV‐DC‐BAR or pFoMV‐DC‐GFP were harvested for total RNA extraction using the RNeasy Plant Mini Kit (Qiagen). The leaves that were sampled are indicated in the figure legends. After first‐strand cDNA synthesis, primer pair 5AmuS2 and 5AmuA2 was used to test for FoMV infection and for the presence of insert. *Z. mays actin* was used as an internal control with primer pair ZmActS and ZmActR.

### 2.5 Herbicide treatment and bar strip test

FoMV‐DP/DC‐BAR‐infected sweet corn plants were sprayed with a dilution of Finale^®^ herbicide that contained 0.05% glufosinate‐ammonium w/v) (BASF) in deionized water. Maize plants were photographed at 10 days after herbicide treatment. Leaf samples of non‐inoculated, pFoMV‐DC‐ or pFoMV‐DC‐BAR‐infected plants were used to test the presence of BAR protein using the QuickStix™ Kit for LibertyLink^®^ *(bar)* Cotton Leaf & Seed (EnviroLogix) according to the manufacturer's instructions.

### 2.6 Microscopy, protein extraction and western blot assays

Leaf samples of non‐inoculated, pFoMV‐DC‐, pFoMV‐DC‐GFP‐infected plants were cut into small squares (1 cm × 0.5 cm) and examined with a Zeiss Axioplan II microscope with FITC cube. Total protein was extracted from leaf samples of non‐inoculated, pFoMV‐DC‐ and pFoMV‐DC‐GFP‐infected and pCAMBIA380‐FoMV‐infected plants. In each sample, 8 leaf disks were collected using a 0.5 cm diameter borer and homogenized in 100 µl of extraction buffer (50 mM NaCl, 20 mM Tris pH 7.5, 1 mM EDTA, 0.1% Triton X‐100, 10% glycerol, 5 mM DTT, 2 mM NaF, 1 mM PMSF, and 1x protease inhibitor cocktail). After centrifugation, the supernatant was mixed with SDS‐PAGE loading buffer and boiled for 5 min, and then, 10 µl of each boiled sample was used in SDS‐PAGE followed by Western blotting using anti‐GFP monoclonal antibody (Genscript).

### 2.7 Insertion of FoMV into a T‐DNA plasmid for agroinoculation

First, the *Xho*I restriction site in pFoMV‐DC was replaced by *Pac*I. To achieve this, a PCR reaction was performed using the primer pair FM‐PacIFor and NosRev with pFoMV‐DC as template. The PCR product was digested with restriction enzymes *Xba*I and *Cla*I, gel extracted, and ligated into pFoMV‐DC that also had been digested with *Xba*I and *Cla*I to produce the pFoMV‐DC‐PacI. Next, the cauliflower mosaic virus 35S promoter to the nopaline synthase terminator was amplified from the pFoMV‐DC‐PacI using primer pair DCPacI1380F and DCPacI1380R. The pCAMBIA1380 backbone was amplified with primer pair 1380F and 1380R. The two PCR products were purified and assembled using the Gibson Assembly^®^ Cloning Kit according to the manufacturer's instructions (New England Biolabs). The fidelity of the resulting pCAMBIA380‐FoMV‐DC* (pFoMV‐DC*) was further confirmed by sequencing. To generate constructs for gRNA delivery, oligonucleotides NbPDSg1 and NbPDSg2 (Table S1) were synthesized and annealed, and then ligated into *Bsu*36I‐ and *Psp*OMI‐digested pFoMV‐DC* to generate the construct pCAMBIA1380‐FoMV‐gNbPDS. The construct pCAMBIA1380‐FoMV‐gZmHKT1 was generated similarly using oligonucleotides ZmHKT1g1 and ZmHKT1g2 (Table S1). The construct pCAMBIA1380‐FoMV‐gSvCA2 was generated by PCR amplifying the sgRNA scaffold with oligonucleotides SvCA2g1 and sgRNA:7xT:PspOMI. This PCR product was purified and assembled into *Bsu*361‐ and *Psp*OMI‐digested pFoMV‐DC* using the Gibson Assembly^®^ Cloning Kit according to the manufacturer's instructions (New England Biolabs).

### 2.8 Agroinfiltration of *N. benthamiana*

*Nicotiana benthamiana* plants between 5 and 6 weeks old were used in this study. *Agrobacterium tumefaciens* strain GV3101 harboring the pFoMV‐DC* plasmids was cultured and re‐suspended in infiltration buffer (200 μM acetosyringone, 10 mM MES, pH 5.6, and 10 mM MgCl_2_) (OD_600_ = 1.0) and kept at room temperature for 3 hr before infiltration into plant leaves with a needleless syringe. Three weeks later, the infected systemic leaves were harvested and used as inoculum for rub‐inoculation onto new sweet corn seedlings at the two‐leaf stage. Three‐leaf stage *S. viridis* seedlings were rub‐inoculated using systemically infected *N. benthamiana* leaves 10 days after infiltration.

For *NbPDS* gene‐editing experiments, *N. benthamiana* transgenic plants expressing the SpCas9 were used. The infiltrated and systemic leaves were sampled at 7, 14, and 21 DPI. Flowers and capsules of infiltrated plants were also sampled to test the *NbPDS* gene editing. In the case of FoMV and TuMV co‐infection, suspensions of GV3101 harboring the pFoMV‐DC*‐gNbPDS and *A. tumefaciens* strain GV2260 harboring the pCB‐TuMV‐GFP (Lellis, Kasschau, Whitham, & Carrington, 2002) were mixed (OD_600_ = 1.0 for each strain, mixed at 1:1) and infiltrated. The infiltrated and systemic leaves were sampled at 7 DPI.

### 2.9 Verification of mutations in *N. benthamiana*, *S. viridis*, and maize

Genomic DNA was extracted from *N. benthamiana* or maize samples using the DNeasy Plant Mini Kit (Qiagen) according to the manufacturer's instructions. The DNA fragments encompassing the target sites were amplified using Q5 High‐Fidelity DNA Polymerase (New England Biolabs) with primer pair NbPDSs and NbPDSa for *NbPDS,* primer pair oEE374 and oEE375 for *SvCA2*, or primer pair ZmIDTF0 and ZmIDTR0 for *ZmHKT1* (Table S1). The *NbPDS* fragment was digested with *Nco*I‐HF and then separated on a 1.0% agarose gel. The intensity of the DNA bands was quantified using ImageJ software to estimate editing efficiency. The undigested bands were individually gel purified and cloned into pGEM‐T Easy (Promega, Madison, WI, USA) for sequence analysis. The *ZmHKT1* and *SvCA2* amplicons were analyzed in a similar way except *Xcm*I and *Pvu*II, respectively, were used for the digestion.

## 3 RESULTS

### 3.1 Construction of FoMV expression vectors

**Figure 1:**
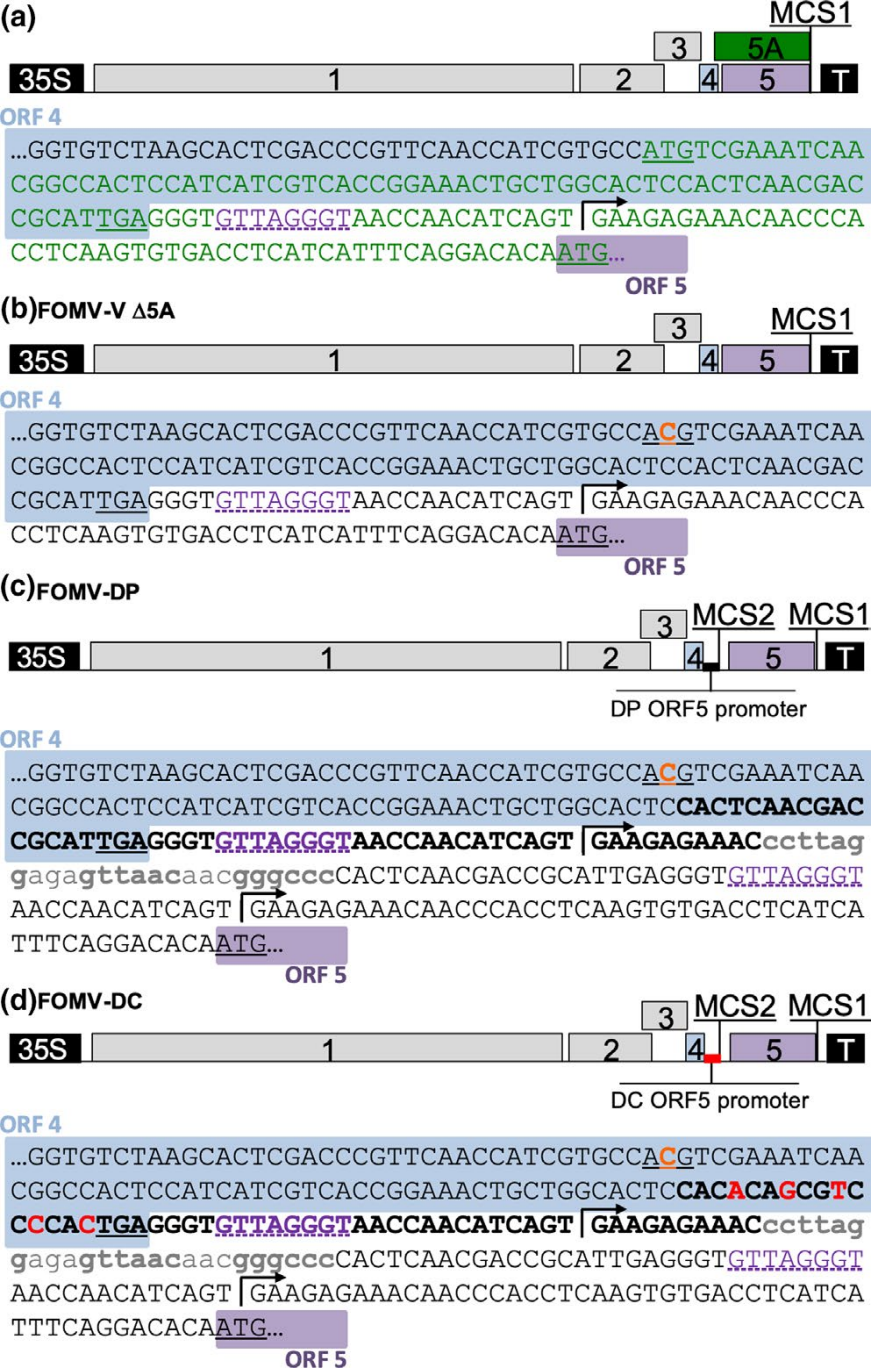
Diagrams of the genomes of FoMV clones and the nucleotide modifications to produce the FoMV‐DP and FoMV‐DC expression vectors. (a) The FoMV‐V silencing vector (pFoMV‐V; Mei et al., 2016) is shown in the upper panel. The lower panel is annotated FoMV sequence beginning in ORF 4 and continuing to the ORF5 start codon. ORF 4 coding sequence is highlighted in blue, ORF 5A is in green text, the predicted core of the ORF 5 promoter is in purple text with a dashed underline, the transcription start site for the ORF 5 subgenomic mRNA transcript is indicated by the black arrow, the beginning of ORF 5 is highlighted in purple, and start or stop codons for all ORFs are indicated by underlining. Multiple cloning site 1 (MCS1) contains the *Xba*I and *Xho*I restriction enzyme sites. (b) FoMV‐V Δ5A in which a point mutation was introduced into the ORF 5A start codon was mutated to render it non‐functional (ATG ‐> ACG) as indicated by the orange text. (c) FoMV‐DP and the sequence showing the duplication of the ORF5 promoter followed by a second multiple cloning site (MCS2). The bold black text indicates the duplicated promoter sequence, and the lowercase gray letters indicate the MCS2 with the *Bsu*36I, *Hpa*I, and *Psp*OMI sites, respectively, in bold. (d) Schematic representation of the FoMV‐DC genome in which the duplicated ORF5 promoter was modified by five point mutations that reduced redundancy of the duplicated sequence. Single nucleotide changes in the duplicated promoter region are in bold red text

**Figure 2:**
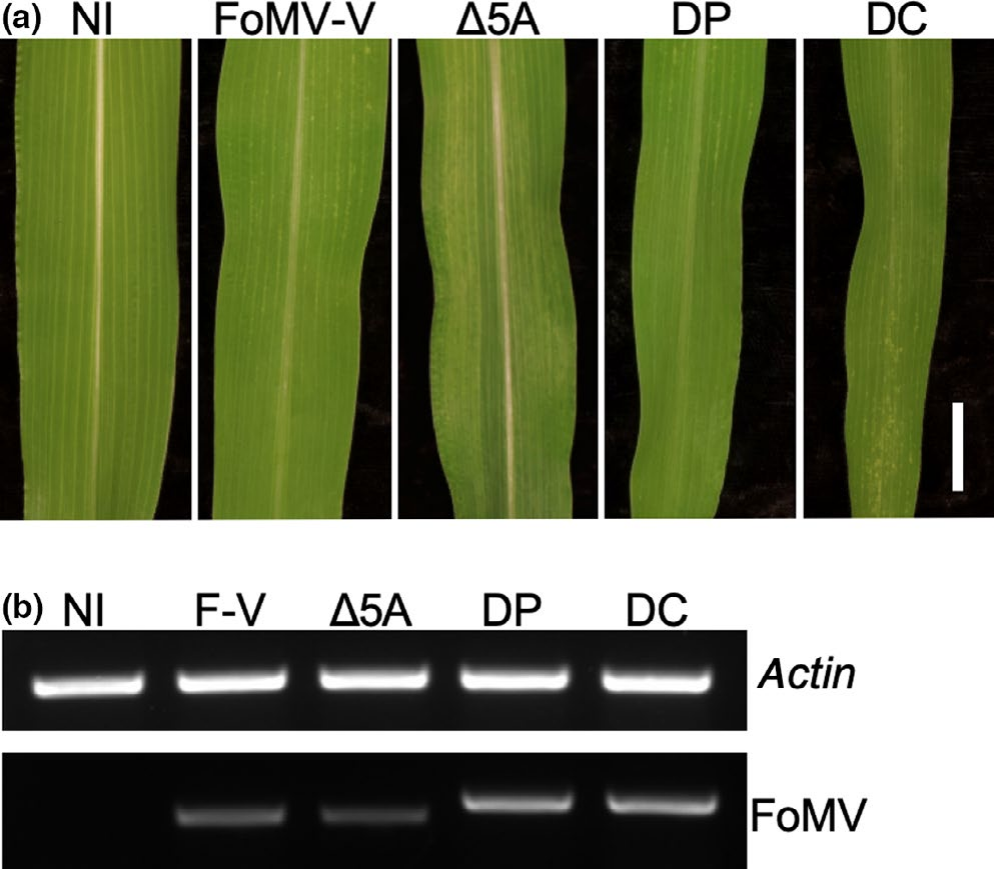
Infection of sweet corn (Golden × Bantam) by the FoMV vectors. (a) Images of leaves from control and inoculated plants. From left to right: non‐inoculated (NI), FoMV silencing vector (FoMV‐V), FoMV‐V with mutated ORF5A (FoMV‐V‐ Δ5A), FoMV expression vectors FoMV‐DP (DP) and FoMV‐DC (DC). Bar = 1 cm. (b) RT‐PCR assay detected FoMV in the systemic leaves shown in panel A. Maize *actin* was included as internal control for RT‐PCR

We previously developed the pFoMV‐V vector for VIGS in maize (Mei et al., 2016), but it cannot be used to express proteins, because the cloning site (MCS1) for plant gene fragments was placed after the stop codon of the CP gene (ORF5) (Figure 1a). To engineer FoMV vectors for protein expression, we designed a CP promoter duplication strategy similar to what has been used in PVX vectors (Dickmeis, Fischer, & Commandeur, 2014; Lacomme & Chapman, 2008; Sablowski, Baulcombe, & Bevan, 1995; Wang et al., 2014) and recently in another FoMV expression vector (Bouton et al., 2018). The CP promoter duplication strategy was potentially complicated by ORF5A, which overlaps ORF4 and ORF5 (Figure 1a). ORF5A was previously shown to be expressed but dispensable for systemic infection in barley (Robertson et al., 2000). To confirm that ORF5A is not required for infection of maize, we made pFoMV‐V Δ5A in which the predicted start codon was changed from ATG to ACG without altering the amino acid sequence of ORF4 (Figure 1b). The infectivity of FoMV‐V Δ5A was tested by both biolistic inoculation and subsequent rub‐inoculation. Plants systemically infected by FoMV‐V Δ5A and FoMV‐V developed mosaic symptoms that were indistinguishable (Figure 2a). The stability of the mutation was tested in 14 biolistically inoculated plants and 13 plants that were rub‐inoculated with sap from a biolistically inoculated plant. The mutation was detected in all the infected samples, and no wild‐type sequence was observed. These results demonstrated that the Δ5A mutation did not revert to wild type and the inability to produce the putative 5A protein did not impair the infectivity of FoMV in maize. Subsequently, pFoMV‐V Δ5A was further modified by introducing two duplicated promoter variants and multiple cloning site 2 (MCS2) containing the *Bsu*36I, *Hpa*I and *Psp*OMI endonuclease cleavage sites. pFoMV‐DP contains a 54‐nucleotide duplication of the CP subgenomic promoter (nucleotides 5280–5333) (Figure 1c), and pFoMV‐DC was made by changing five codons in the sequence that overlaps with ORF4 without altering the encoded amino acid sequence (Figure 1d).

Sweet corn seedlings infected with pFoMV‐DP or pFoMV‐DC developed mosaic symptoms indistinguishable from the parental pFoMV‐V or pFoMV‐V Δ5A clones (Figure 2a). RT‐PCR was used to amplify a 396‐nucleotide fragment containing the duplicated promoter and MCS2. The RT‐PCR product was detected in symptomatic sweet corn plants that were biolistically inoculated with pFoMV‐V, pFoMV‐V Δ5A, pFoMV‐DP, or pFoMV‐DC but not in non‐inoculated control plants (Figure 2b). The slightly larger band detected in FoMV‐DP‐ or FoMV‐DC‐infected leaves suggested that the duplicated promoter remained intact during maize infection (Figure 2b). We also confirmed the authenticity of subgenomic RNAs expressed by using 5’ RACE to map the transcription start sites for the duplicated CP promoter and the authentic CP promoter. This analysis verified that the duplicated CP promoter produced subgenomic mRNA transcripts that initiated 14 nucleotides downstream of the core promoter sequence at a GAA sequence as does the authentic CP promoter (Figure 1).

### 3.2 Expression of the Bialaphos Resistance (BAR) protein from FoMV

**Figure 3:**
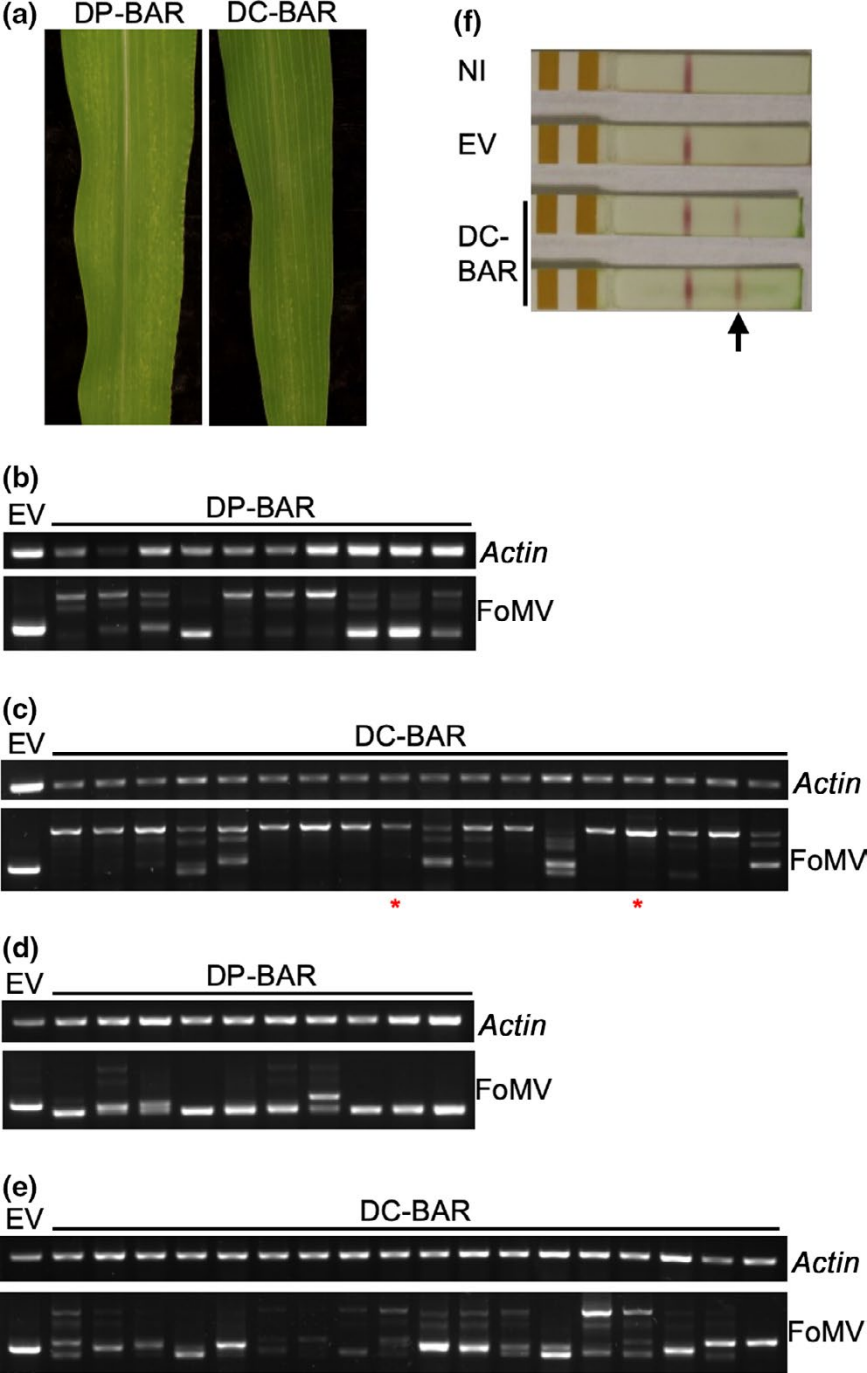
FoMV‐mediated BAR expression in sweet corn (Golden × Bantam). (a) Sweet corn inoculated with pFoMV‐DP‐BAR (DP‐BAR, left) and pFoMV‐DC‐BAR (DC‐BAR, right). (b) RT‐PCR analysis of *bar* insert stability in the 4th leaves of FoMV‐DP‐BAR‐infected plants. (c) RT‐PCR analysis of *bar* insert stability in the 4th leaves of FoMV‐DC‐BAR‐infected plants. (d) RT‐PCR analysis of *bar* insert stability in the 6th leaves of FoMV‐DP‐BAR‐infected plants. (e) RT‐PCR analysis of *bar* insert stability in the 6th leaves of FoMV‐DC‐BAR‐infected plants. The upper gel images in (b, c, d, and e) are the RT‐PCR control showing amplification of a maize *actin* mRNA fragment in all samples. The lower gel images are RT‐PCR amplification across the FoMV cloning site (MCS2). EV indicates the FoMV‐DP or DC empty vector that carries no insert. (F) Strip test for the expression of BAR protein. Positive signals indicated by the arrow are detected in FoMV‐DC‐BAR‐infected leaf tissue, but not in leaves of non‐infected (NI) or FoMV‐DC empty vector (EV) control plants. The red stars in panel C indicate the plants infected with FoMV‐DC‐BAR that were used in the strip test

To test protein expression, the *bar* gene was inserted into pFoMV‐DP and pFoMV‐DC to produce pFoMV‐DP‐BAR and pFoMV‐DC‐BAR, respectively. Expression of BAR was expected to protect plants from the herbicide Finale^®^ (BASF) (Whitham, Yamamoto, & Carrington, 1999), which has glufosinate‐ammonium as the active compound. Within ten days after biolistic inoculation with pFoMV‐DP‐BAR and pFoMV‐DC‐BAR, 50%–90% of inoculated plants developed typical mosaic symptoms starting from the 3rd leaf (e.g., Figure 3a). To confirm infection and test stability of the insertion, RNA was extracted from the 4th and 6th leaves of 10 FoMV‐DP‐BAR and 18 FoMV‐DC‐BAR plants for RT‐PCR analysis using FoMV primers that flanked the cloning site. A 475‐bp PCR product was expected in the plants infected by the empty vectors, and a 1,027‐bp product in the plants infected by FoMV‐DP‐BAR or FoMV‐DC‐BAR. In the leaf 4 samples, the 1,027‐bp PCR product was detected in 9 out of 10 FoMV‐DP‐BAR plants, and all of them also contained smaller deletion derivatives (Figure 3b). The 1,027‐bp product was detected in 17 out of 18 FoMV‐DC‐BAR plants, and 6 of them also contained smaller deletion derivatives (Figure 3c). The deletion of BAR was more extensive in the 6th leaf samples in which all the FoMV‐DP‐BAR and FoMV‐DC‐BAR plants carried partial to total deletions (Figure 3d,e). Overall, the *bar* insert appeared to be less stable in FoMV‐DP than in the FoMV‐DC context, which was expected since the FoMV‐DC vector was designed to reduce redundancy with the native CP promoter. Expression of the BAR protein was confirmed by an immunoassay in leaf four of two plants infected by FoMV‐DC‐BAR (Figure 3f).

**Figure 4:**
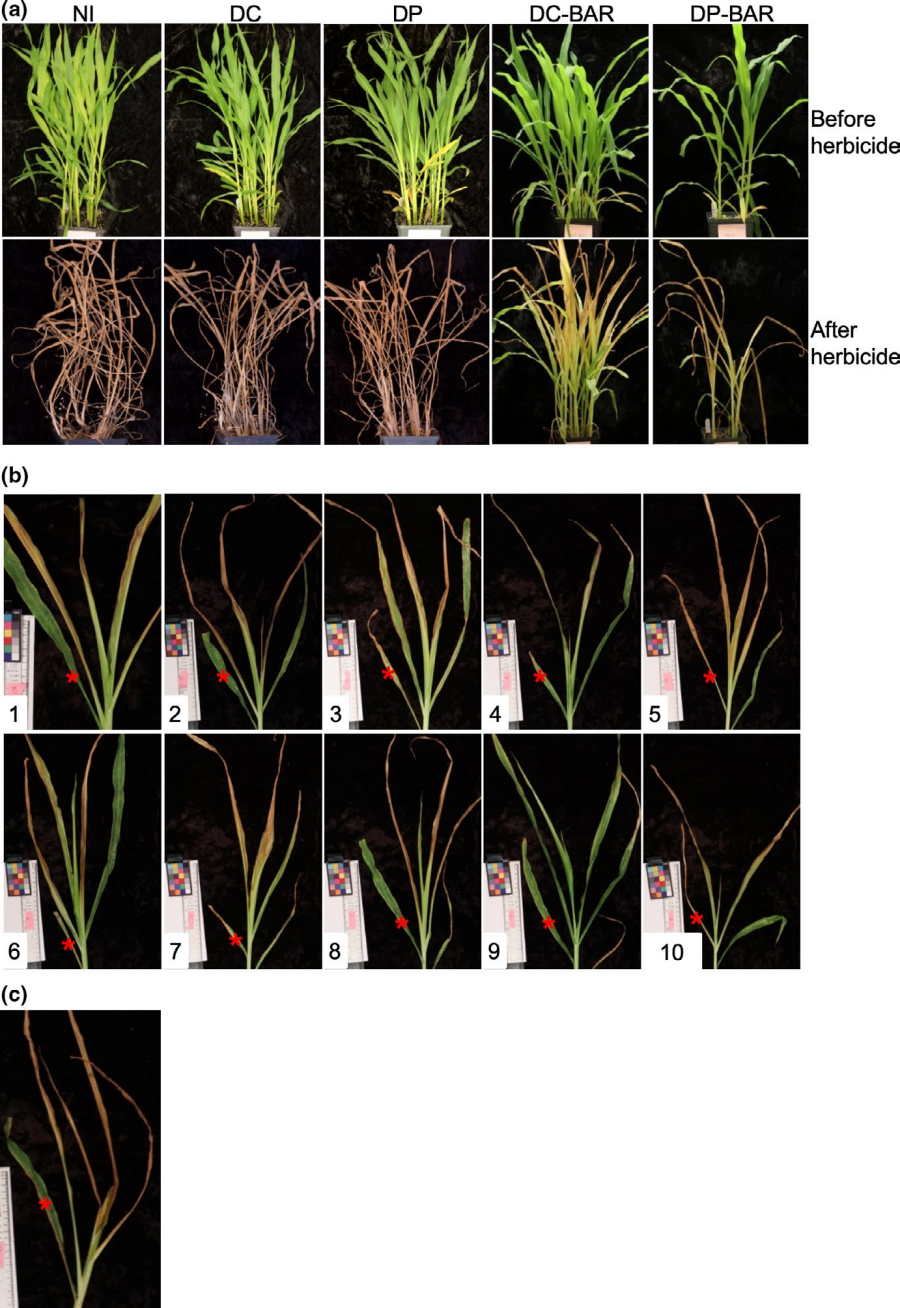
Expression of BAR from FoMV transiently partially protects sweet corn plants from herbicide treatment. (a) Sweet corn plants before (upper panels) and after (lower panels) treatment with glufosinate herbicide. Plants were photographed at 18 dpi, just prior to herbicide treatment, and at 10 days after the first herbicide application. From left to right: non‐inoculated (NI), plants infected with pFoMV‐DC (DC), pFoMV‐DP (DP), pFoMV‐DC‐BAR (DC‐BAR), and pFoMV‐DP‐BAR (DP‐BAR). (b) Individual DC‐BAR plants after herbicide treatment. (c) A representative image of a DP‐BAR plant after herbicide treatment. Red stars in b and c indicate the 4th leaves

To test whether the expression of BAR could provide herbicide resistance, plants were sprayed with 0.05% glufosinate‐ammonium twice at a 3‐day interval and then scored for response at 10 days after the first application. The herbicide treatment killed the non‐infected, FoMV‐DP, and FoMV‐DC plants, but plants infected by FoMV‐DP‐BAR and FoMV‐DC‐BAR were partially or totally protected (Figure 4a). The protective effect varied among plants, but the 4th leaf remained green on most of the plants (Figure 4b,c). When the herbicide was applied at a later stage beginning at 23 dpi with pFoMV‐DP‐BAR and pFoMV‐DC‐BAR, all plants were killed (Figure S1), which is consistent with the extensive deletion of the *bar* gene in leaf 6 (Figure 3d,e).

To test whether the expression of BAR could be passaged, the sap from leaves of biolistically inoculated FoMV‐DC‐BAR plants with confirmed BAR expression (Figure 3f) was used to rub‐inoculate the first two leaves of healthy sweet corn seedlings. Mosaic symptoms were observed in 25 of 30 plants approximately one week after rub‐inoculation. These plants and corresponding controls were treated with 0.05% glufosinate‐ammonium at 13 DPI. At 10 days after herbicide treatment, 16 FoMV‐DC‐BAR plants were protected with at least one leaf remaining green, and all leaves remained green on 8 of the plants (Figure S2). These data showed that functional BAR protein was expressed when leaf sap from pFoMV‐DC‐BAR‐inoculated plants was rub‐inoculated onto new sweet corn plants, and the protective effect is similar in biolistic and rub‐inoculated plants.

### 3.3 Expression of GFP from FoMV

**Figure 5:**
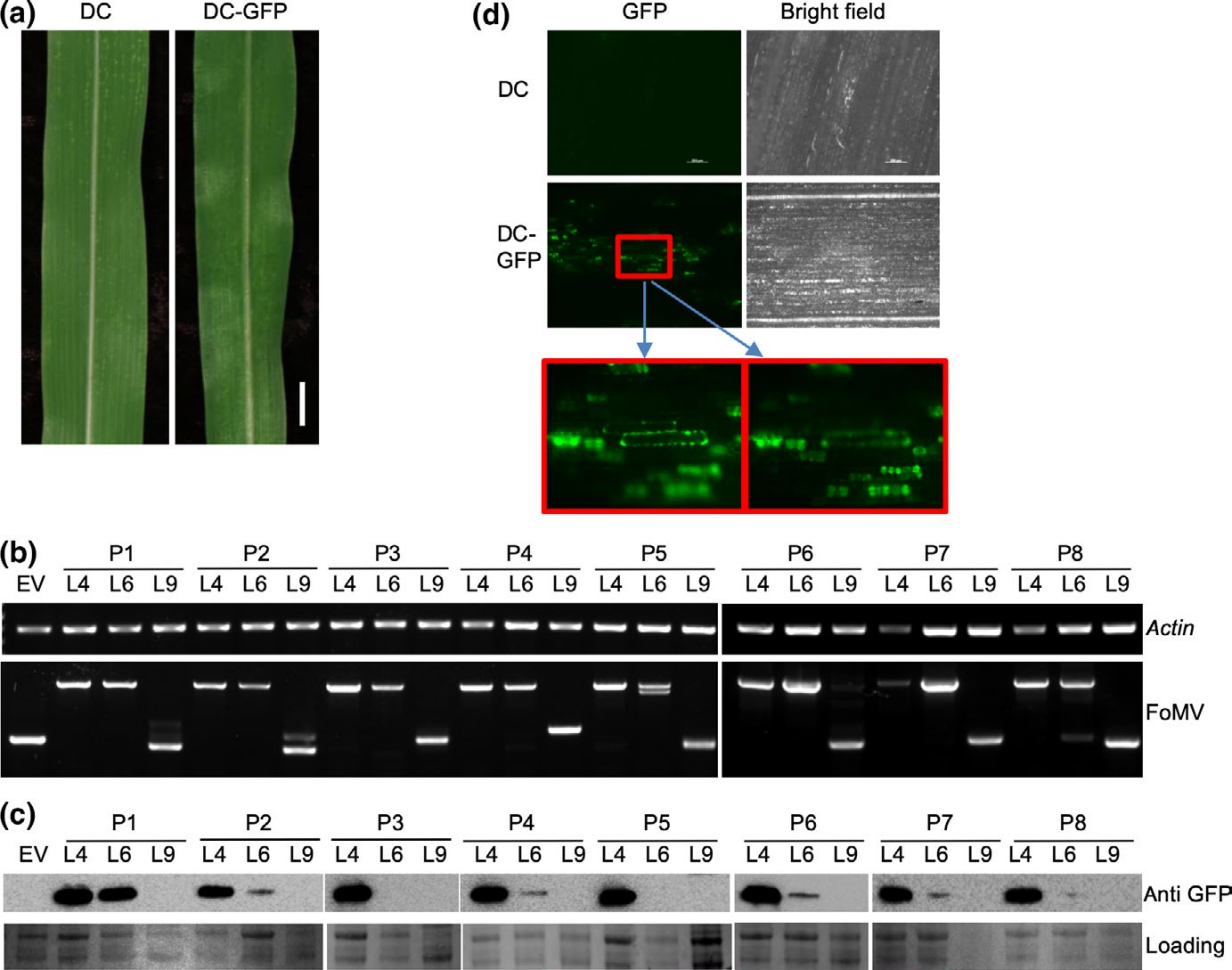
Expression of GFP from FoMV in sweet corn. (a) Sweet corn inoculated with pFoMV‐DC (DC, left) and by pFoMV‐DC‐GFP (DC‐GFP, right). (b) RT‐PCR analyses for the *GFP* insert stability in FoMV‐DC‐GFP‐infected plants. The upper gel image is the RT‐PCR control showing amplification of a maize *actin* mRNA in all samples. The lower gel image shows RT‐PCR amplification across the FoMV cloning site MSC2. EV indicates the FoMV‐DC empty vector that carries no insert. L4, L6, and L9 indicate the leaf number that was sampled. (c) Immunoblot assay showing GFP expression in FoMV‐DC‐GFP‐infected leaf tissues that are used in panel B. The upper images are immunoblots using anti‐GFP antibody, and the lower images show the protein loading control. (d) Green fluorescence in leaf 4 of plants infected with FoMV‐DC or FoMV‐DC‐GFP. The area in the red box is enlarged in the lower panels, which are in two different focal planes to illustrate GFP fluorescence in epidermal (left) and mesophyll cells (right)

The *GFP* sequence was inserted at the *Bsu36*I and *PspOM*I cloning sites in pFoMV‐DC to produce pFoMV‐DC‐GFP. GFP expression was not investigated using pFoMV‐DP, because *bar* was more stable in pFoMV‐DC. Sweet corn seedlings were inoculated with pFoMV‐DC‐GFP using biolistic bombardment, and mosaic symptoms developed that were indistinguishable from pFoMV‐DC‐inoculated plants (Figure 5a). The presence and stability of the *GFP* insert was tested by RT‐PCR analysis using primers that flanked the cloning site. A 475‐bp PCR product was expected in plants infected by FoMV‐DC, and an 1,192‐bp PCR product was expected in plants infected by FoMV‐DC‐GFP. No deletion of GFP was detected in leaf 4 samples, because we only observed the 1,192‐bp band, and we saw evidence for a deletion only in the leaf 6 sample of plant 5 (Figure 5b; P5). However, the 1,192‐bp band was not detected in any of the leaf 9 samples, indicating that no intact FoMV‐DC‐GFP remained (Figure 5b).

Expression of GFP protein was analyzed by Western blot using an anti‐GFP antibody. Consistent with the RT‐PCR results, GFP was detected in all the leaf 4 samples and most of the leaf 6 samples but not in any of the leaf 9 samples (Figure 5c). In addition, bands from the leaf 4 samples were the most intense, indicating that GFP accumulated to the highest levels in leaf 4 relative to leaf 6. The leaves were examined using a fluorescence microscope to detect GFP fluorescence. Green fluorescence was observed in epidermal and mesophyll cells of leaves infected by FoMV‐DC‐GFP but not in control leaves infected by FoMV‐DC (Figure 5d). The green fluorescence occurred in a patchy manner, reflecting the symptoms of FoMV infection (Figure 5d). The GFP signals were relatively strong in leaf 4 and became weaker as the virus moved to upper leaves and were not detectable in the 9th leaf (Figure S3).

### 3.4 Development of a FoMV vector compatible with agroinoculation

**Figure 6:**
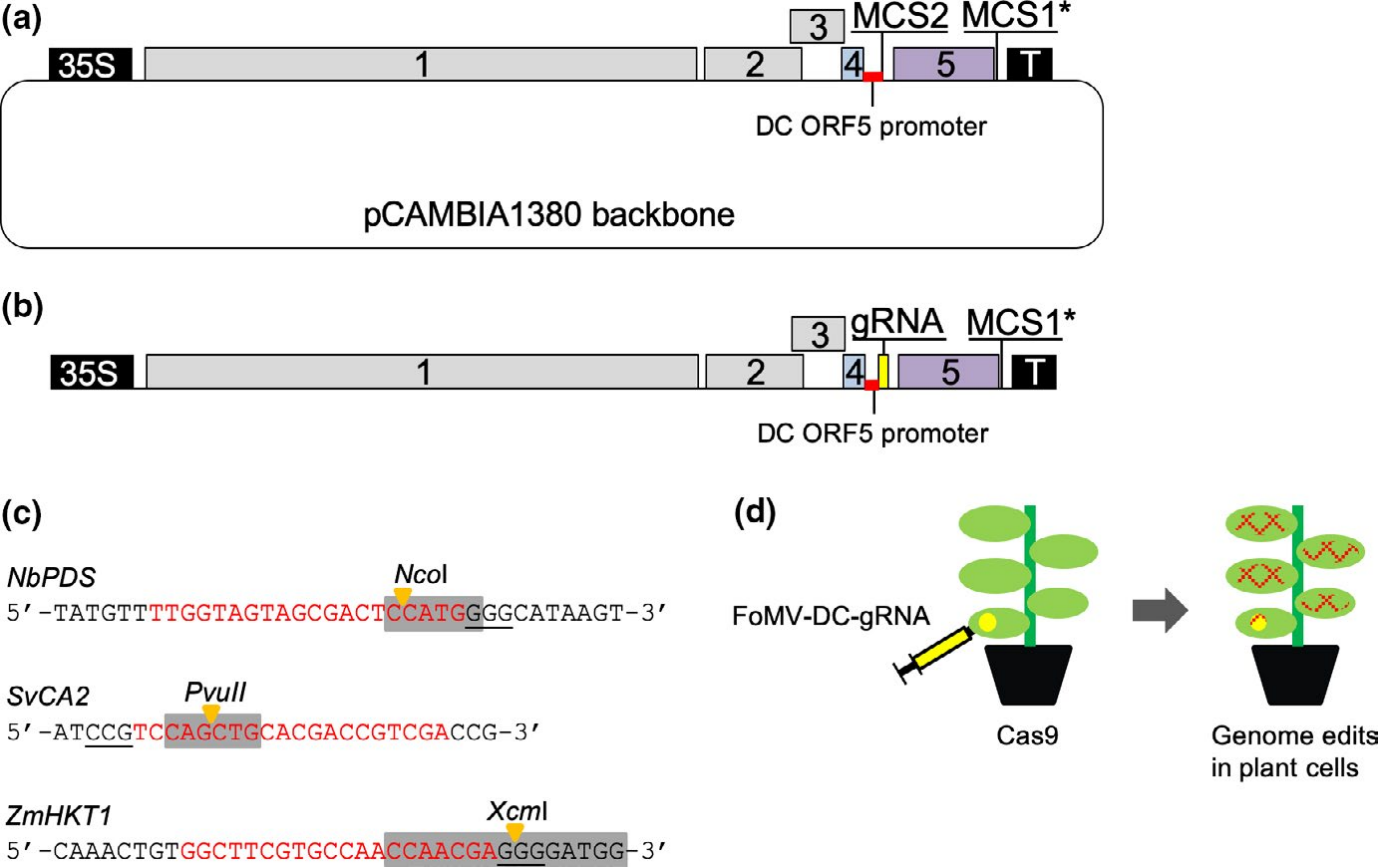
FoMV agroinoculation and expression of guide RNAs. (a) Schematic representation of FoMV vector in the pCAMBIA1380 binary vector. MCS1* indicates that the MCS1 site was modified to *Xba*I and *Pac*I. (b) The yellow box indicates the position of insertion of a single 20‐nucleotide guide RNA sequence fused to the 83‐nucleotide scaffold (gRNA) at MCS2 of pFoMV‐DC*. (c) gRNA target sites for *N. benthamiana Pds1 S. viridis CA2*, and *Z. mays HKT1*. Red text indicates the 20‐nucleotide gRNA sequence, the gray boxes indicate the recognition sites for *Nco*I, *Pvu*II and *Xcm*I restriction endonucleases, the yellow arrows indicate the cleavage sites for the restriction endonucleases, and the protospacer adjacent motifs are indicated by the underlined text. (d) Strategy to express gRNA from the pFoMV‐DC* vector in plants expressing the Cas9 protein. The red cross‐hatch indicates that gene edits were expected to be observed in inoculated and systemic leaves in which FoMV‐DC*‐gRNA is accumulating

FoMV can infect *N. benthamiana*, which is inoculated easily by Agrobacterium infiltration (agroinoculation), a much simpler method than biolistic inoculation. To facilitate agroinoculation, the CaMV 35S promoter, genome of FoMV‐DC, and nopaline synthase terminator were amplified as a single fragment from pFoMV‐DC and inserted into the binary T‐DNA vector pCAMBIA1380 (Figure 6a). The *XhoI* restriction endonuclease site at MCS1 was not unique in the pCAMBIA1380 context, so it was replaced with *Pac*I to create MCS1*. The infectivity of pCAMBIA1380‐FoMV‐DC* was then tested by agroinoculation of *N. benthamiana* plants. Mild mosaic symptoms were observed on systemic *N. benthamiana* leaves at approximately 3 weeks after inoculation (Figure S4a). Sweet corn seedlings were subsequently rub‐inoculated with sap from FoMV‐infected *N. benthamiana* leaves, and typical mosaic symptoms were observed within a week (Figure S4b). FoMV infection in the agroinoculated *N. benthamiana* plants and rub‐inoculated sweet corn plants was confirmed by Western blot using anti‐FoMV‐CP antibody (Figure S4c). These results demonstrated that the pCAMBIA1380‐FoMV‐DC* (pFoMV‐DC*) was infectious through agroinoculation and could be passaged by rub‐inoculation to maize.

### 3.5 FoMV‐mediated Gene Editing in *N. benthamiana*

**Figure 7:**
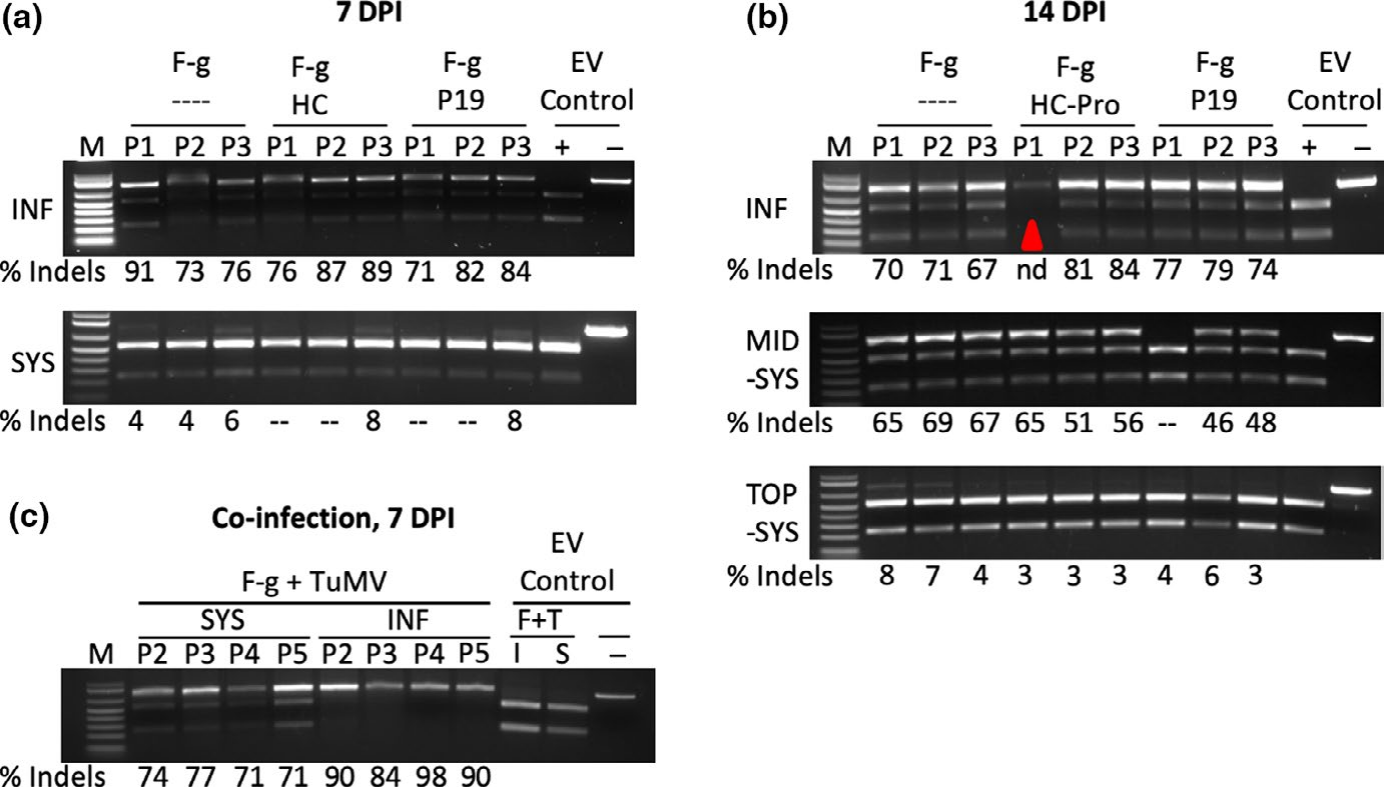
FoMV‐mediated editing of *NbPDS* in agroinfiltrated and systemic leaves of *N. benthamiana* plants expressing Cas9. (a) PCR‐based assay to detect edits in *NbPDS* at 7 days post‐inoculation (DPI) in agroinfiltrated (INF) and systemic (SYS) leaves. Oligonucleotide primers were used to generate 797‐bp amplicons flanking the *NbPDS* guide RNA target site, and the amplicons were incubated with *Nco*I. The wild‐type amplicons are cleaved into two bands of 541 bp and 256 bp, amplicons carrying edits that disrupt the *Nco*I site are not cleaved. F‐g, FoMV‐DC* expressing the *NbPDS* guide RNA; HC, co‐infiltration with a construct expressing the TuMV helper component‐proteinase; P19, co‐infiltration with a construct expressing the TBSV 19 kDa protein; M, DNA size marker; P#, plant number; % Indels, percent of each PCR amplicon that was not digested; ‐‐, indicates that a non‐digested product was not detected and therefore was not quantified; +, wild‐type amplicon cleaved by *Nco*I; ‐, wild‐type amplicon that was not incubated with *Nco*I. (b) PCR‐based assay to detect edits in *NbPDS* at 14 DPI in agroinfiltrated (INF) and systemic (SYS) leaves. The systemic leaves were subdivided into two categories: middle systemic (MID‐SYS) and top systemic (TOP‐SYS). The “nd” and the red triangle indicate the sample for which indels were not quantified and sequence not determined due to inefficient PCR. (c) PCR‐based assay to detect edits in *NbPDS* at 7 DPI in agroinfiltrated (INF) and systemic (SYS) leaves during a co‐infection of FoMV‐DC*‐gNbPDS and turnip mosaic virus (TuMV). F + T, FoMV‐DC* empty vector + TuMV; I, agroinfiltrated; S, systemic

To test whether single gRNA produced from FoMV‐DC* can mediate gene editing, we inserted a gRNA targeting the *N. benthamiana Phytoene desaturase* gene (*NbPDS*) at MCS2 to produce the pFoMV‐DC*‐gNbPDS construct (Figure 6b,c). This gRNA was previously reported to mediate gene editing when expressed from tobacco rattle virus in *N. benthamiana* expressing Cas9 (Ali et al., 2015). The gRNA target site overlaps a recognition site for the *Nco*I restriction endonuclease (Figure 6c). *N. benthamiana* plants expressing Cas9 (Baltes et al., 2015) were agroinoculated with pFoMV‐DC*‐gNbPDS (Figure 6d). Genomic DNA was extracted from infiltrated and systemic leaves sampled at 7, 14, and 21 DPI to test modification of *NbPDS*. PCR was used to produce 797‐bp amplicons containing the target sequence that were then digested with *Nco*I (Figure 7). After *Nco*I digestion, the wild‐type amplicon yielded two bands of 541 bp and 256 bp, but many of the amplicons from FoMV‐DC*‐gNbPDS leaves yielded an additional band of approximately 797 bp that was resistant to *Nco*I digestion (Figure 7). The presence of the non‐digested band suggested that the target sequence was altered. At 7 DPI, the percentage of the modified DNA was estimated in the range of 73%‐91% in the infiltrated leaves and from 0% to 8% in the top systemic leaves (Figure 7a). To confirm that the *NbPDS* gene was modified at the gRNA target site, the *Nco*I‐resistant bands were cloned and sequenced. The sequences confirmed that the *Nco*I site was disrupted mostly by small deletions (1 to 23 bp) that initiated 3 bp upstream of the PAM (Figure S5). At 14 DPI, we collected samples from the infiltrated leaves, top systemic leaves that were asymptomatic at the time, and the middle systemic leaves that displayed viral symptoms. Similar to the 7 DPI samples, there was much more non‐digested amplicon in the infiltrated leaves compared to the top leaves (Figure 7b). Interestingly, the amount of non‐digested amplicon was similar in the middle systemic leaves and the infiltrated leaves. Sequence analysis of the undigested amplicons showed a similar spectrum of small indels occurring within 3bp of the PAM (Figure S6).

To test whether editing could be observed in flowers, a set of plants was inoculated and flowers were collected beginning at approximately 2 months after inoculation. *NbPDS* gene editing was detected in the flower tissues of FoMV‐DC*‐gNbPDS‐infected Cas9‐overexpressing plants with the percent of indels based on *Nco*I digestion ranging from 6.9% to 14.8% (Figure S7). These data demonstrated that FoMV continued to express functional guide RNAs over the course of growth and development in *N. benthamiana*. Seed was collected from capsules derived from the two flowers above those that were collected for DNA extraction and sown in soil. We did not observe any photobleached (albino) seedlings, which was the expected phenotype if *NbPds* loss‐of‐function mutations were inherited (Ali et al., 2015).

To test whether the efficiency of FoMV‐mediated *NbPDS* gene editing could be elevated in the presence of viral silencing suppressors, we first co‐infiltrated the Cas9 *N. benthamiana* with a mixture of *Agrobacterium* strains carrying pFoMV‐DC*‐gNbPDS and p35S‐HcPro or pCB‐P19. We observed similar amounts of the non‐digested band in infiltrated and systemic leaves at 7 and 14 DPI when compared to pFoMV‐DC*‐gNbPDS alone (Figure 7a,b), suggesting that the localized expression of the silencing suppressors did not enhance editing. Next, we co‐infiltrated pFoMV‐DC*‐gNbPDS with an infectious clone of turnip mosaic virus (TuMV) expressing GFP (pCB‐TuMV‐GFP) (Lellis et al., 2002). TuMV is a potyvirus that systemically infects *N. benthamiana*, and because it carries HcPro, we expected it to interact synergistically with FoMV and increase the frequency of editing in systemic tissues. This TuMV‐GFP clone is highly virulent on *N. benthamiana* plants and, in our conditions, causes plants to die at about 10 DPI, so infiltrated leaves and top systemic leaves were only sampled at 7 DPI. The co‐infection elevated the estimated percentage of indels in systemic leaves from 4%–6% to >70%, which was comparable to the infiltrated leaves in the FoMV‐DC*‐gNbPDS single infection (Figure 7c; Figure S8). As observed for the single FoMV‐DC*‐gNbPDS experiments, the gene edits were characterized by small deletions ranging in size from 1 to 11 bp (Figure S9).

To further test the effect of TuMV HcPro, the Cas9 *N. benthamiana* plants were crossed with TuMV HcPro *N. benthamiana* plants. F1 plants were genotyped for presence of Cas9 and HcPro and then inoculated with pFoMV‐DC*‐gNbPDS (Figure S10). The percent of indels in systemic leaves was 2%–4% in the absence of HcPro and 6%–37% in the presence of HcPro. This result indicates that the TuMV HcPro silencing suppressor promotes gene editing, although the effect is variable. The plants were grown to maturity, and all seeds from the first two pods produced on each plant were sown in soil. We expected to observe albino seedlings if any carried heritable *NbPDS* loss‐of‐function mutations (Ali et al., 2015), but there were none, which indicates that FoMV‐DC*‐gNbPDS did not induce heritable edits in *N. benthamiana*.

### 3.6 FoMV‐mediated gene editing in *S. viridis*

**Figure 8:**
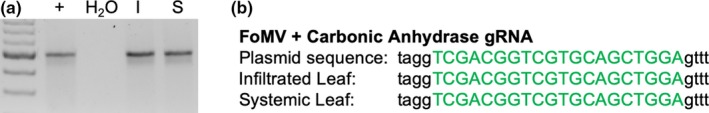
Guide RNAs (gRNAs) are stably maintained at MCS2 in the FoMV‐DC* vector. A single guide RNA (gRNA) targeting *S. viridis Carbonic anhydrase 2* (*SvCA2*, Sevir.5G247900) fused to the 83‐nucleotide tracrRNA scaffold was inserted into MCS2. (a) *SvCA2* gRNA stability was tested in *S. viridis* plants. The FoMV‐DC*‐gSvCA2 construct was infiltrated into *N.* *benthamiana* and rub‐inoculated onto young *S. viridis* leaves. The agarose gel shows the presence of stable inserts in *S viridis* leaves. + indicates a plasmid control, I indicates the inoculated leaf, and S indicates an upper systemic leaf. (b) Sanger sequencing of the RT‐PCR bands from *S. viridis* inoculated with FoMV‐DC*‐gSvCA2. The insert is maintained after passage through *N. benthamiana* into *S.* *viridis* leaves without acquiring any deletions or mutations

**Figure 9:**
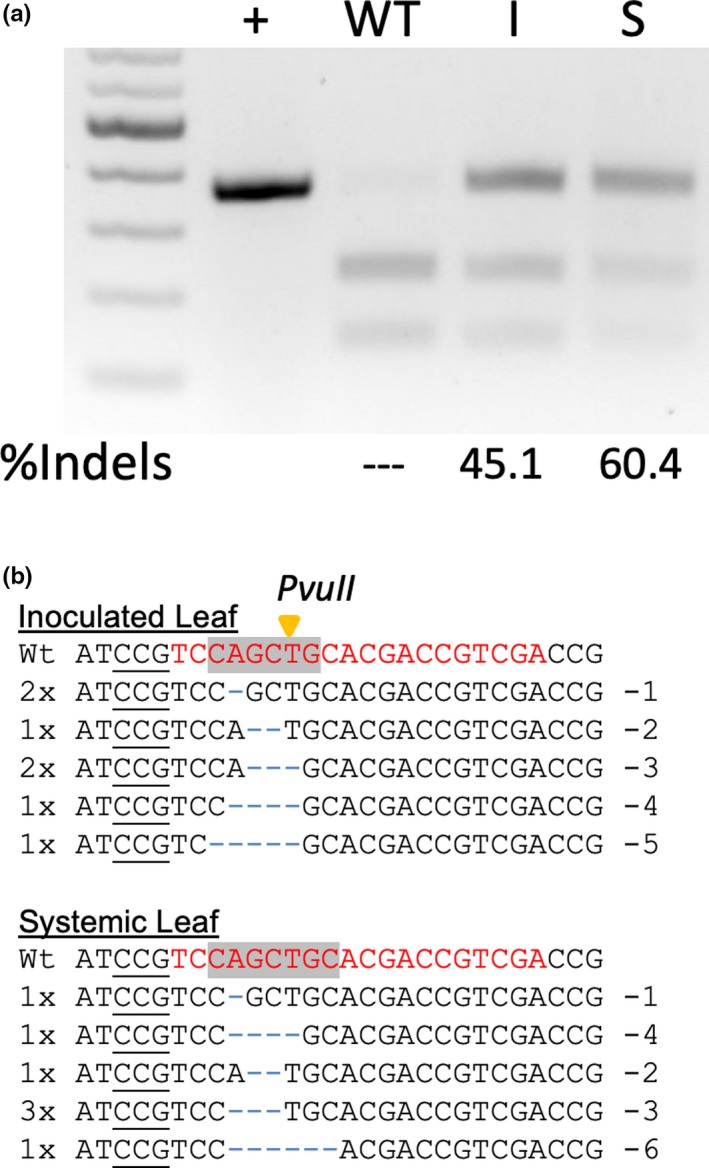
FoMV‐mediated editing of *SvCA2* in systemic leaves of *S. viridis* plants expressing Cas9. (a) PCR‐based assay to detect edits in *SvCA2* at 14 days post‐inoculation in an upper systemic leaf. FoMV‐gRNA vectors were propagated in *Nicotiana benthamiana*, and at 10 days after infiltration, a systemic leaf was used to rub‐inoculate Cas9 *S. viridis* plants. Oligonucleotide primers were used to generate 360‐bp amplicons flanking the *SvCA2* guide RNA target site for multiple infected plants. The amplicons were incubated with *Pvu*II restriction enzyme to create wild‐type cleaved bands of 228 bp and 132 bp. Amplicons carrying edits that disrupt the *PvuII* site are not cleaved. The gel shown here represents a plant with successful disruption of the *PvuII* target site by the FoMV‐gRNA vector. + indicates a plasmid control, I indicates the inoculated leaf, and S indicates an upper systemic leaf. % Indels, percent of each PCR amplicon that was not digested as measured by ImageJ software; ‐‐, indicates that a non‐digested product was not detected and therefore was not quantified. (b) Sequence analysis of the non‐cleaved amplicons that were gel purified and cloned. The *PvuII* recognition site is indicated by the gray box in the wild‐type sequence, and the cleavage site is indicated by the yellow arrow. The protospacer adjacent motif (PAM) is indicated by the underlined sequence, and the guide RNA sequence is indicated by the red text. The blue dashes represent nucleotide deletions. Each sample is from an individual clone, negative numbers to the right of a sequence show the number of nucleotides deleted, and (#x) shows the number of times that sequence occurred

To test FoMV‐mediated gRNA delivery in monocots, we used a gRNA that targets the first exon of *Carbonic anhydrase 2* (*SvCA2*, Sevir.5G247900) of *S. viridis* (Figure 6b). The FoMV‐DC*‐gSvCA2 clone was first agroinoculated onto *N. benthamiana* to create inoculum that was rub‐inoculated onto a *S. viridis* line expressing Cas9. To test gRNA stability, RT‐PCR analysis was performed on inoculated and systemic leaf tissues. The gRNA insertion was detected in both the inoculated and systemic leaves, and sequence analysis confirmed that the gRNA remained intact following passage from *N. benthamiana* to *S. viridis* (Figure 8a,b). To determine whether gene edits occurred in the inoculated or systemic *S. viridis* leaves, DNA was extracted and used as a template to PCR amplify the target region in non‐inoculated plants, inoculated leaves and systemic leaves. A 360‐bp amplicon that is cleaved into bands of 228 bp and 132 bp by *Pvu*II is expected in the wild type, but if the *Pvu*II site is disrupted due to gene editing, then a mixture of cleaved and uncleaved amplicons is expected. Infection by FoMV‐DC*‐gSvCA2 resulted in about 45% indels in the inoculated leaf and 60% indels in the systemic leaf (Figure 9a). The non‐digested amplicons were cloned and sequenced, and we observed a series of small deletions (1–6 bp) that had occurred within 2–4 bp of the PAM sequence, disrupting the *Pvu*II cleavage site (Figure 9b). These data demonstrate that FoMV delivered a functional guide RNA that could mediate Cas9‐directed cleavage of *SvCA2*. Progeny of the plants infected with FoMV‐DC*‐gSvCA2 did not carry edits in *SvCA2* indicating that FoMV did not induce heritable edits in *S. viridis.*

### 3.7 FoMV‐mediated gene editing in maize

**Figure 10:**
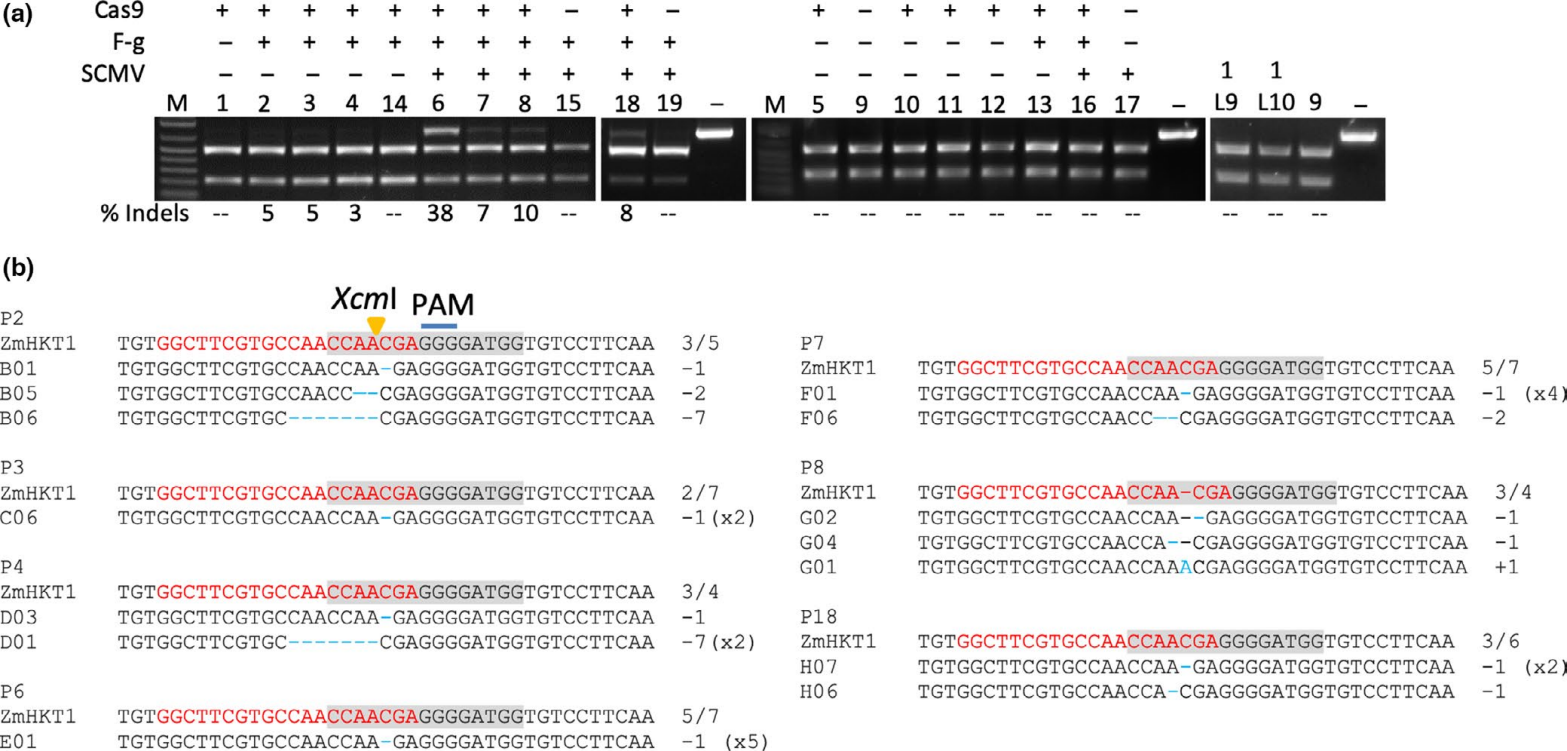
FoMV‐mediated editing of *ZmHKT1* in systemic leaves of maize plants expressing Cas9. (a) PCR‐based assay to detect edits in *ZmHKT1* systemic leaf 9. Oligonucleotide primers were used to generate 732‐bp amplicons flanking the *ZmHKT1* guide RNA target site, and the amplicons were incubated with *Xcm*I. The wild‐type amplicons are cleaved into two bands of 504 bp and 228 bp, amplicons carrying edits that disrupt the *Xcm*I site are not cleaved. RT‐PCR was used to determine the presence of the Cas9 transgene, FoMV‐DC*‐gZmHKT1 (F‐g), and sugarcane mosaic virus (SCMV) in each leaf sample. The numbers above each lane indicate the plant from which the leaf sample was derived. M, DNA size marker; % Indels, percent of each PCR amplicon that was not digested; ‐‐, indicates that a non‐digested product was not detected and therefore was not quantified; 1L9, a leaf 9 sample from plant 1; 1L10, a leaf 10 sample from plant 1; ‐, wild‐type amplicon that was not digested. (b) Sequence analysis of amplicons that were gel purified and cloned. The *Xcm*1 recognition site is indicated by the gray box in the wild‐type sequence, and the cleavage site is indicated by the yellow arrow. The protospacer adjacent motif (PAM) is indicated by the blue line, and the guide RNA sequence is indicated by the red text. The blue dashes represent nucleotide deletions, blue letters represent nucleotide insertions, and a red dash indicates a gap in the alignment to the guide RNA sequence due to a nucleotide insertion in one of the amplicon sequences. P# indicates the corresponding plant number, and the ratio to the right of the wild‐type sequences indicate the number of clones that carried an indel out of the total number of clones sequenced for that plant sample. Negative numbers to the right of a sequence show the number of nucleotides deleted, positive numbers show the number of nucleotides inserted, and (x#) shows the number of times that sequence occurred

To test gene editing in maize, a gRNA targeting *ZmHKT1* was inserted into pFoMV‐DC* to create pFoMV‐DC*‐gZmHKT1. The *ZmHKT1* gRNA targets a sequence overlapping a recognition site for the *Xcm*I endonuclease (Figure 6c) (Xing et al., 2014). *Xcm*I digestion of the wild‐type 732‐bp PCR amplicon containing the target sequence yielded two bands of 504 bp and 228 bp. Seedlings from a maize line segregating for Cas9 (Char et al., 2017) were co‐inoculated with FoMV‐DC*‐gZmHKT1 and the potyvirus SCMV, which expresses an HcPro (Zhang et al., 2008). Co‐infection of FoMV and SCMV enhances FoMV accumulation and results in more severe disease symptoms (Figure S11), which is typical of potyvirus synergisms (Pruss, Ge, Shi, Carrington, & Bowman Vance, 1997; Shi, Miller, Verchot, Carrington, & Vance, 1997). Presence of the Cas9 transgene mRNA was detected using RT‐PCR, and the viruses were detected by Western blot using anti‐FoMV‐CP and anti‐SCMV‐CP antibodies (Figure 10). Genomic DNA was extracted from leaf 9 of the plants, and amplicons containing the *ZmHKT1* target site were produced by PCR and then incubated with *Xcm*I. Only amplicons produced from plants that were Cas9 positive and infected by FoMV‐DC*‐gZmHKT1 or FoMV‐DC*‐gZmHKT1 + SCMV were partially resistant to *Xcm*I digestion. We estimated that 3%–6% of the amplicons from Cas9 + FoMV‐DC*‐gZmHKT1 contained indels that disrupted the *Xcm*I site and 7%–38% of the amplicons from Cas9 + FoMV‐DC*‐gZmHKT1 + SCMV contained indels that disrupted the *Xcm*1 site (Figure 10a). However, not all plants that were positive for Cas9 and FoMV‐DC*‐gZmHKT1 alone or in combination with SCMV produced amplicons resistant to *Xcm*I (see plants 14, 13, and 16; Figure 10a). As expected, no non‐digested amplicons were detected in either Cas9‐negative or FoMV‐DC*‐gZmHKT1‐negative plants (see plants 1, 15, 5, 9, 11, 12, 17; Figure 10a). Similar to *N. benthamiana* and *S. viridis*, small deletions within two to three bp of the PAM were most common in maize (Figure 10b). We were not able to obtain seeds from these plants due to growth chamber conditions and the severe symptoms of co‐infection with SCMV. Therefore, the possibility of heritable mutations in *ZmHKT1* could not be tested.

## 4 DISCUSSION

Here, we describe a set of recombinant FoMV vectors that have applications for gene expression and single gRNA delivery, and they were demonstrated to function in maize, *S. viridis*, and *N. benthamiana*. These vectors were derived from a previous FoMV vector developed by us for VIGS, which could not be used for gene expression because of the position of MCS1 after the stop codon of ORF5 (Mei et al., 2016). Our new vectors can be inoculated into plants using either DNA particle bombardment or agroinfiltration. Although VIGS was not tested using the new vector set, they are expected to be useful for this purpose, given a previous demonstration of VIGS using a duplicated CP promoter strategy in FoMV by Liu et al. (2016). In order to enable foreign gene expression, the subgenomic promoter duplication strategy, which has been successfully demonstrated in PVX vectors (Dickmeis et al., 2014; Lacomme & Chapman, 2008; Sablowski et al., 1995; Wang et al., 2014), was adopted to generate pFoMV‐DP/DC vectors. A similar strategy was used in another FoMV expression vector developed by Bouton et al. (2018), although different approaches were taken to design and build the vectors.

To engineer the pFoMV‐DP/DC vectors, we first confirmed that the ORF5A was dispensable for infection of our primary target plant, maize. Robertson et al. (2000) previously showed that ORF5A is not required for systemic infection of barley plants. ORF5A starts 144 nucleotides upstream of and is in frame with the CP ORF, and it is predicted to produce a variant of CP with a 48‐amino acid N‐terminal extension (Bruun‐Rasmussen et al., 2008; Robertson et al., 2000). Disrupting ORF5A simplified the duplicated CP cloning strategy for the expression of proteins and allowed us to reduce the duplicated CP promoter region to 54 nucleotides. We aimed to minimize the size of the duplication, while retaining the promoter function, to reduce the risk of recombination (Dickmeis et al., 2014). We confirmed that the expected subgenomic mRNA transcripts initiating at 14 nucleotides downstream of the core promoter sequence at GAA were produced from the duplicated CP subgenomic promoter by using 5’ RACE. In the FoMV‐DC vectors, the duplicated nucleotides overlapping with ORF4 were further modified by changing the third base of each codon, which yielded better stability of the *bar* insertion. It may be possible to further stabilize expression from FoMV using a heterologous promoter strategy as shown for PVX by Dickmeis et al. (2014) and for other viruses, such as TMV (Roy et al., 2011). However, this approach may not be straightforward, because the 8 nucleotide CP subgenomic promoter core sequence differs at two positions relative to most other characterized potexviruses (Kim & Hemenway, 1997), and the FoMV CP subgenomic promoter did not function in the context of the PVX genome (Dickmeis et al., 2014).

To test gene expression, the coding sequences of the BAR (552 bp) and GFP (720 bp) marker proteins were inserted into the FoMV‐DC and FoMV‐DP vectors. Although expression varied from plant to plant, when inoculated at the two‐leaf stage, protein expression was observed from L4 to L6. In the case of GFP expression, green fluorescence was detected through L8 and no fluorescence signal was observed in L9 samples, which was consistent with the results of RT‐PCR showing total loss of the *GFP* insertion in all L9 samples. Our previous work found that when plants were infected by the FoMV vector carrying 200–300 bp insertions at MCS1 after the ORF5 stop codon, plants began to show evidence for deletions of the inserts by L9 and partial or total deletion was observed in L top (leaf 12–14) (Mei et al., 2016). Considering that the insertion size of *bar* and *GFP* in FoMV‐DC and FoMV‐DP is approximately twice as big, an earlier and more extensive deletion was not surprising. In line with a relationship between insert stability and size, we found that single gRNA, which are 103 nucleotides in size, was very stable at MCS2 in both the FoMV‐DC and FoMV‐DP contexts, with no evidence for deletions in *S. viridis* or maize plants. This influence of insert size is in agreement with observations in PVX vectors (Avesani et al., 2007).

The most exciting aspect of this work is a demonstration that FoMV can deliver functional gRNA in monocot plants, maize and *S. viridis*, expressing Cas9 and in *N. benthamiana*. We tested proof of concept in *N. benthamiana* by agroinoculation where FoMV‐DC*‐gNbPDS induced indels at a high frequency in the agroinoculated leaf patches. Indels were also observed in the systemic leaves at differing frequencies depending on the timing and location of the sampled leaves. To enhance the frequency of editing in systemic tissues, viral silencing suppressors were provided by transient agroinfiltrations, co‐infections, and stable transformation. Transient agroinfiltration of HcPro and P19 did not enhance editing, because this localized method of delivery could not promote FoMV accumulation in systemic tissues. Co‐inoculation of FoMV with the potyvirus, TuMV, resulted in remarkably high levels of gene edits in systemic leaves at 7 dpi, but plants died within a few days, so further analyses were not possible. The high frequency of editing was in stark contrast with the low frequency of editing in comparable systemic leaves of plants that had been inoculated with FoMV‐DC*‐gNbPDS alone. These results indicate that TuMV and FoMV interact synergistically, likely due to the HcPro silencing suppressor carried by TuMV (Kasschau et al., 2003). The increased frequency of editing is expected to be due to increased accumulation of FoMV genomic and subgenomic RNAs resulting in more production of guide RNAs. In addition, the suppression of silencing may also enhance expression of the Cas9 transgene. Additional experiments will be required to distinguish among these possibilities. Consistent with the idea that HcPro may have promoted these effects, inoculation of plants expressing both Cas9 and HcPro with FoMV‐DC‐gNbPDS also resulted in a higher, but variable frequency of gene editing in systemic leaves compared to plants expressing Cas9 alone. HcPro's ability to promote gene editing is consistent with results from Mao et al. (2018) who showed that the tomato bushy stunt P19 silencing suppressor improved gene editing by the CRISPR/Cas9 system in *Arabidopsis thaliana*.

To extend this idea further, we co‐inoculated maize seedlings segregating for Cas9 with FoMV‐DC*‐gZmHKT1 and SCMV. The presence of SCMV promoted FoMV accumulation and resulted in more severe disease than with either virus alone, which is consistent with a synergistic interaction mediated by a potyvirus. Consistent with TuMV, co‐infection of SCMV with FoMV‐DC*‐gZmHKT1 resulted in the highest levels of gene edits in systemic leaves, although the frequency was variable. Taken together, we conclude that synergistic viruses or their silencing suppressors can greatly enhance FoMV‐mediated gene editing. However, SCMV + FoMV co‐infection results in severe symptoms that prevent proper maize development, and so phenotypic effects of edits would be difficult to discern. An important line of investigation for the future will be to optimize silencing suppressor activity to enable more efficient gene editing without such severe symptoms.

The vector set and experiments presented here demonstrate that recombinant FoMV clones have a wide variety of potential uses for VIGS, VOX, and VEdGE (virus‐enabled gene editing). The utility of FoMV for these different applications can supplement conventional genetics and transgenic plant approaches for identifying gene functions in maize, or to investigate the functions of novel proteins in maize. Our work demonstrates that there are limitations when performing studies using VOX, because of the stability of inserts, but we expect that FoMV can be useful for expressing proteins that can induce phenotypes in leaves 4–6 of young maize plants. The single gRNA inserts appear to be stable, and so FoMV could be used to induce gene edits in vegetative tissues of monocot plants expressing Cas9. Based on the frequency of indels in maize plants expressing Cas9, we do not expect to be able to observe phenotypes due to gene editing.

Heritable gene edits were not observed in the progeny of *N. benthamiana* or *S. viridis* plants. In *N. benthamiana*, edits were observed in the flowers, but they must have occurred in maternal tissues, such as sepals and petals. The lack of heritable edits is consistent with the inability of FoMV to be seed transmitted in *S. viridis* (Paulsen & Niblett, 1977). Because single guide RNA can vary in their efficiency and specificity (Doench et al., 2016), the ability of FoMV to induce gene edits in monocot vegetative tissues is expected to have useful applications for rapidly screening single gRNA candidates prior to producing gRNA‐Cas9 transgenic lines carrying heritable edits. A very interesting possibility is that Cas9 and gRNA could be expressed simultaneously from the FoMV genome as was recently shown for the negative strand cytorhabdovirus, BYSMV, which can tolerate insertion of the 4.4 kb Cas9 coding sequence and a single gRNA (Gao et al., 2019). The recombinant BYSMV could induce gene edits in the agroinfiltrated leaves of *N. benthamiana* plants, but systemic gene editing was not reported. We expect that additional work will be needed to stabilize an ORF as large as Cas9 in the FoMV genome, so that it could be used to express both Cas9 and gRNA for systemic gene editing in maize and other monocots.

## CONFLICT OF INTEREST

The authors declare no conflict of interest associated with the work described in this manuscript.

## AUTHOR CONTRIBUTIONS

Y.M., E.K., D.F.V., and S.A.W. conceived the original research plans; D.F.V. and S.A.W. supervised the experiments; Y.M., B.M.B., E.E.E., and E.K. performed most of the experiments; A.K.N. provided critical materials and guidance; and Y.M., B.M.B., E.E.E., D.F.V., and S.A.W. wrote the article with contributions of all the authors. S.A.W. agrees to serve as the author responsible for contact and ensures communication.

## RESPONSIBILITIES OF THE AUTHOR FOR CONTACT

It is the responsibility of the author for contact to ensure that all scientists who have contributed substantially to the conception, design, or execution of the work described in the manuscript are included as authors, in accordance with the guidelines from the Committee on Publication Ethics (COPE) (http://publicationethics.org/resources/guidelines). It is the responsibility of the author for contact also to ensure that all authors agree to the list of authors and the identified contributions of those authors. The author responsible for contact and ensuring the distribution of materials integral to the findings presented in this article in accordance with the Journal policy described in the Instructions for Authors (http://www.plantphysiol.org) is S. A. Whitham (swhitham@iastate.edu).
