# Supplementary material for: Protein expression and gene editing in monocots using foxtail mosaic virus vectors
Source: Plant Direct. 2019 Nov 22;3(11):e00181. doi: 10.1002/pld3.181 (PMC6874699; doi:10.1002/pld3.181)
Supplement: Supplementary file 1 [file PLD3-3-e00181-s001.pdf]

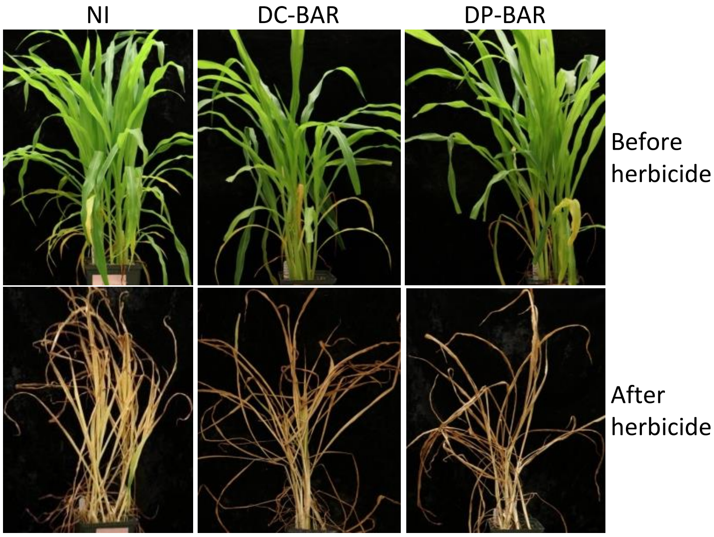

Supplemental Figure 1. Sweet corn plants before (upper panels) and after (lower panels) treatment of Finale herbicide starting at 23 DPI. From left to right: non-inoculated (NI), plants infected by FoMV-DC-BAR (DC-BAR) and FoMV-DP-BAR (DP-BAR).
