# Supplementary material for: Protein expression and gene editing in monocots using foxtail mosaic virus vectors
Source: Plant Direct. 2019 Nov 22;3(11):e00181. doi: 10.1002/pld3.181 (PMC6874699; doi:10.1002/pld3.181)
Supplement: Supplementary file 2 [file PLD3-3-e00181-s002.pdf]

A

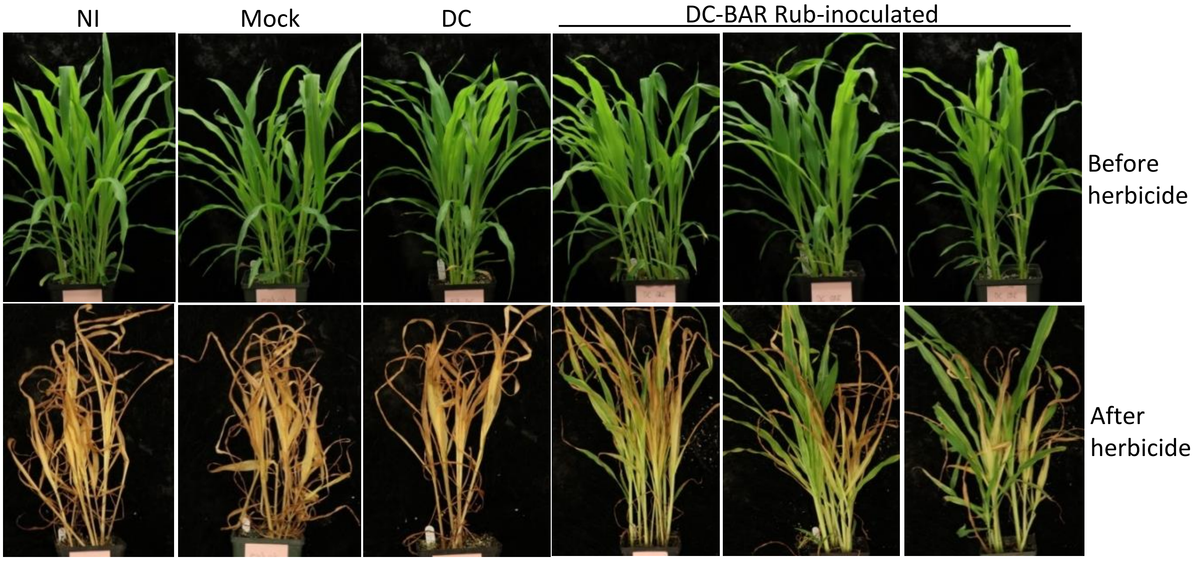

B

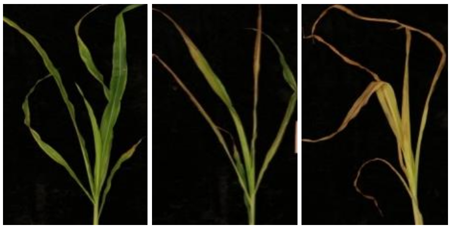

Supplemental Figure 2. FoMV-DC-BAR partially and transiently protects sweet corn plants from Finale herbicide. **A.** Sweet corn plants before (upper panels) and after (lower panels) treatment of Finale herbicide starting at 13 DPI. From left to right: non-inoculated (NI), mock treated (Mock), plants rub-inoculated by FoMV-DC (DC EV) and plants rub-inoculated by FoMV-DC-BAR (DC BAR). **B.** Representative images of DC-BAR rub-inoculated plants after herbicide treatment. From left to right: green, partial green and yellow plant.
