# Supplementary material for: Protein expression and gene editing in monocots using foxtail mosaic virus vectors
Source: Plant Direct. 2019 Nov 22;3(11):e00181. doi: 10.1002/pld3.181 (PMC6874699; doi:10.1002/pld3.181)
Supplement: Supplementary file 3 [file PLD3-3-e00181-s003.pdf]

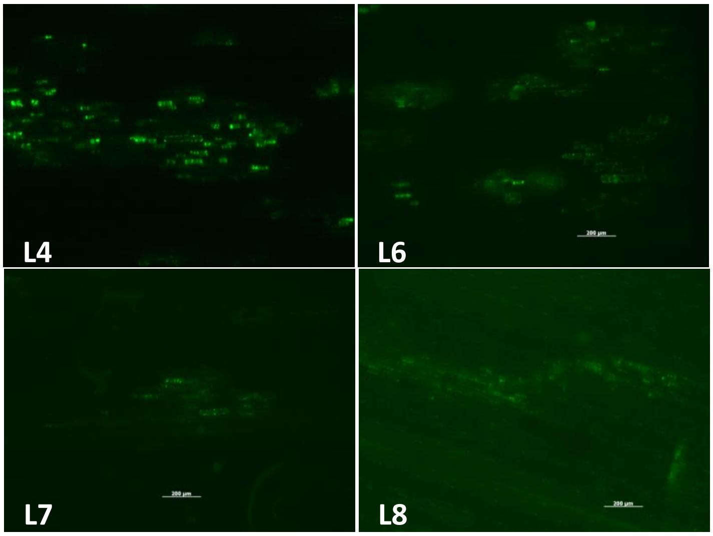

Supplemental Figure 3. Green fluorescence observed in leaves of sweet corn plants infected with FoMV-DC-GFP. L4, L6, L7 and L8 indicate the leaf number that was imaged. The bar = 200 µm.
