# Supplementary material for: Protein expression and gene editing in monocots using foxtail mosaic virus vectors
Source: Plant Direct. 2019 Nov 22;3(11):e00181. doi: 10.1002/pld3.181 (PMC6874699; doi:10.1002/pld3.181)
Supplement: Supplementary file 4 [file PLD3-3-e00181-s004.pdf]

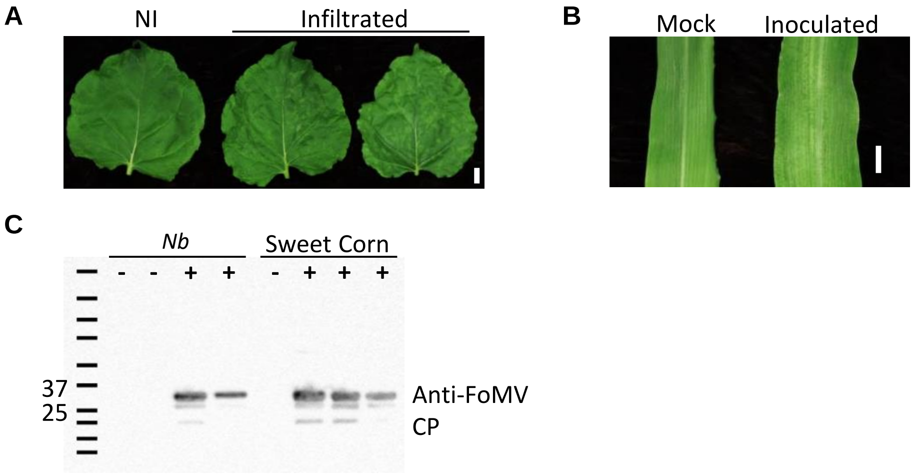


**Supplemental Figure 4.** Confirmation of infectivity of the pCAMBIA1380-FoMV-DC*. **A.** Systemic leaf of *Nicotiana benthamiana* (*Nb*) plants that were non-inoculated (NI) or agroinfiltrated with pCAMBIA-FoMV-DC empty vector. Bar=1 cm. **B.** Mock-inoculated leaf and systemic leaf from a plant that was rub-inoculated with sap from a *Nb* plant that had been agroinoculated with pCAMBIA1380-FoMV-DC*. Bar=1 cm. **C.** A western blot showing the accumulation of FoMV capsid protein in systemic leaves of Nb and sweet corn plants that are shown in panels **A** and **B**. '–' indicates NI or mock treated; + indicates leaf with viral symptoms.
