# Supplementary material for: Protein expression and gene editing in monocots using foxtail mosaic virus vectors
Source: Plant Direct. 2019 Nov 22;3(11):e00181. doi: 10.1002/pld3.181 (PMC6874699; doi:10.1002/pld3.181)
Supplement: Supplementary file 5 [file PLD3-3-e00181-s005.pdf]

```
                                        NcoI    PAM
PDS-I1
NbPDS    TATGTTTTGGTAGTAGCGACTCCATGGGGCATAAGT   3/3
A10      TATGTTTTGGTAGTAGCGACTC-ATGGGGCATAAGT   -1(x2)
B10      TATGTTTTGGTAG----------ATGGGGCATAAGT   -10

PDS-I2
NbPDS    TATGTTTTGGTAGTAGCGACTCCA-TGGGGCATAAGT  6/8
E02      TATGT------------------------TCATAAGT   -23
C02      TATGTTTTGGTAGC---------A-TGGGGCATAAGT   -9
H02      TATGTTTTGGTAGTAG-------A-TGGGGCATAAGT   -7
G02      TATGTTTTGGTAGTAGCGAC---A-TGGGGCATAAGT   -2
A02      TATGTTTTGGTAGTAGCGACTC-A-TGGGGCATAAGT   -1
B02      TATGTTTTGGTAGTAGCGACTCCAATGGGGCATAAGT   +1A

PDS-I3
NbPDS    TATGTTTTGGTAGTAGCGACTCCATGGGGCATAAGT   4/4
D03      TATGTTTTGGTAGTAGCGACTC-ATGGGGCATAAGT   -1(x3)
A03      TATGTTTTGGTAGTAG-------ATGGGGCATAAGT   -7

PDS+HcPro-I1
NbPDS    TATGTTTTGGTAGTAGCGACTCCATGGGGCATAAGT   4/4
E03      TATGTTTTGGTAGTAGCGACTC-ATGGGGCATAAGT   -1(x2)
F03      TATGTTTTGGTAGTAGCGACT--ATGGGGCATAAGT   -2
H03      TATGTTTTGGTAGTAGCGAC---ATGGGGCATAAGT   -3

PDS+HcPro-I2
NbPDS    TATGTTTTGGTAGTAGCGACTCCATGGGGCATAAGT   3/3
A04      TATGTTTTGGTAGTA--------ATGGGGCATAAGT   -8
B04      TATGTTTTGGTAGTAGCGACTC-ATGGGGCATAAGT   -1
C04      TATGTTTTGGTAGTAG-------ATGGGGCATAAGT   -7

PDS+HcPro-I3
NbPDS    TATGTTTTGGTAGTAGCGACTCCATGGGGCATAAGT   2/3
D04      TATGTTTTGGTAG----------ATGGGGCATAAGT   -10
E04      TATGTTTTGGTAGTAGCGACTC-ATGGGGCATAAGT   -1

PDS+P19-I1
NbPDS    TATGTTTTGGTAGTAGCGACTCC-ATGGGGCATAAGT  6/8
A05      TATGTTTTGGTAGTAGCGACT-C-ATGGGGCATAAGT  -1(x3)
B05      TATGTTTTGGTAGTAGCGA---C-ATGGGGCATAAGT  -3
C05      TATGTTTTGGTAGTAGCGACTCCCATGGGGCATAAGT  +1(x2)

PDS+P19-I2
NbPDS    TATGTTTTGGTAGTAGCGACTCCATGGGGCATAAGT   1/1
G04      TATGTTTTGGTAGTAGCG-----ATGGGGCATAAGT   -5

PDS+P19-I3
NbPDS    TATGTTTTGGTAGTAGCGACTCC-ATGGGGCATAAGT  6/8
D06      TATGTTTTGGTAGTAGCGACT-C-ATGGGGCATAAGT  -1(x2)
C06      TATGTTTTGGTAGTAGCGA---C-ATGGGGCATAAGT  -3(x2)
G06      TATGTTTTGGTAGTAGCGACTCCCATGGGGCATAAGT  +1(x2)

PDS-S1
NbPDS    TATGTTTTGGTAGTAGCGACTCCATGGGGCATAAGT   3/8
C07      TATGTTTTGGTAGTAGCGAC---ATGGGGCATAAGT   -3
G07      TATGTTTTGGTAGTAGCGAC---ATGGGGCATAAGT   -7(x2)

PDS+P19-S3
NbPDS    TATGTTTTGGTAGTAGCGACTCCATGGGGCATAAGT   1/8
F09      TATGTTTTGGTAGTAGCGAC---ATGGGGCATAAGT   -3
```

**Supplemental Figure 5.** FoMV-DC*-gNbPDS induced edits recovered from *Cas9 N. benthamiana* at 7 days post inoculation. Sequence analysis of amplicons that were gel purified and cloned. The *Nco*1 recognition site is indicated by the gray box in the wild type (*NbPDS*) sequence and the cleavage site is indicated by the yellow arrow. The proto-spacer adjacent motif (PAM) is indicated by the blue line, and the guide RNA sequence is indicated by the red text. Blue dashes represent nucleotide deletions, blue letters represent nucleotide insertions, and a red dash indicates a gap in the alignment to the guide RNA sequence due to a nucleotide insertion in one of the amplicon sequences. PDS-I# samples are from the leaves infiltrated with FoMV-DC*-gNbPDS. PDS+HCPro-I# samples are from the leaves co-infiltrated with FoMV-DC*-gNbPDS and 35S::HC-Pro. PDS+P19-I# samples are from leaves co-infiltrated with FoMV-DC*-gNbPDS and 35S::P19. PDS-S1 and PDS+P19-S3 are samples from systemic leaves of plants from which the corresponding infiltrated leaf samples were taken. The ratio to the right of the wild type sequences indicate the number of clones that carried an indel out of the total number of clones sequenced for that plant sample. Negative numbers to the right of a sequence show the number of nucleotides deleted, positive numbers show the number of nucleotides inserted, and (x#) shows the number of times that sequence occurred if more than once.
