# Supplementary material for: Protein expression and gene editing in monocots using foxtail mosaic virus vectors
Source: Plant Direct. 2019 Nov 22;3(11):e00181. doi: 10.1002/pld3.181 (PMC6874699; doi:10.1002/pld3.181)
Supplement: Supplementary file 6 [file PLD3-3-e00181-s006.pdf]

```
PDS-I1                                      NcoI   PAM
NbPDS    TATGTTTTGGTAGTAGCGACTCCATGGGCATAAGT   3/5
B01      TATGTTTTGGTAGTAGC------ATGGGGCATAAGT -6
C01      TATGTTTTGGTAGTAGCGAC---ATGGGGCATAAGT -3
D01      TATGTTTTGGTAGTAGCGACTCC-----GCATAAGT -5

PDS-I2
NbPDS    TATGTTTTGGTAGTAGCGACTCC-ATGGGGCATAAGT 6/8
E08      TATGTTTTGGTAGTAGCGACTCCAATGGGGCATAAGT +1
C08      TATGTTTTGGTAGTAGCGACT-C-ATGGGGCATAAGT -1(X2)
G08      TATGTTTTGGTAGTAGCGA---C-ATGGGGCATAAGT -3
A08      TATGTTTTGGTAGTAG------C-ATGGGGCATAAGT -6
D08      TATGTTTTGGTAGTAGC----------GGGCATAAGT -9

PDS-I3
NbPDS    TATGTTTTGGTAGTAGCGACTCCATGGGGCATAAGT  2/7
B09      TATGTTTTGGTAGTAGC------ATGGGGCATAAGT -6
H09      TATGTTTTGGTAGT---------ATGGGGCATAAGT -9

PDS+HcPro-I2
NbPDS    TATGTTTTGGTAGTAGCGACTCC-ATGGGGCATAAGT 5/5
D02      TATGTTTTGGTAGTAGCGACTCCCATGGGGCATAAGT +1
A02      TATGTTTTGGTAGTAGCGACT-C-ATGGGGCATAAGT -1(X2)
B02      TATGTTTTGGTAGTAGCGAC----ATGGGGCATAAGT -3
C02      TATGTTTTGGTAGTAG--------ATGGGGCATAAGT -7

PDS+HcPro-I3
NbPDS    TATGTTTTGGTAGTAGCGACTCC-ATGGGGCATAAGT 8/8
D10      TATGTTTTGGTAGTAGCGACTCCAATGGGGCATAAGT +1
F10      TATGTTTTGGTAGTAGCGACTCCCATGGGGCATAAGT +1
A10      TATGTTTTGGTAGTAGCGACT-C-ATGGGGCATAAGT -1(X4)
C10      TATGTTTTGGTAGTAGC---------TGGGCATAAGT -7
H10      TATGTTTTGGTAGTAG--------ATGGGGCATAAGT -7

PDS+P19-I1
NbPDS    TATGTTTTGGTAGTAGCGACTCC-ATGGGGCATAAGT 5/6
A03      TATGTTTTGGTAGTAGCGACTCCAATGGGGCATAAGT +1
D03      TATGTTTTGGTAGTAGCGACTCCCATGGGGCATAAGT +1(X2)
B03      TATGTTTTGGTAGTAGCGAC----ATGGGGCATAAGT -3
C03      -----------------------------GCATAAGT -49

PDS+P19-I2
NbPDS    TATGTTTTGGTAGTAGCGACTCCATGGGGCATAAGT  3/5
A04      TATGTTTTGGTAGTAGCGACTC-ATGGGGCATAAGT -1(X2)
C04      TATGTTTTGGTAGTAG-------ATGGGGCATAAGT -7

PDS+P19-I3
NbPDS    TATGTTTTGGTAGTAGCGACTCCATGGGGCATAAGT  5/7
E11      TATGTTTTGGTAGTAGCGACTC-ATGGGGCATAAGT  -1
G11      TATGTTTTGGTAGTAGCGAC---ATGGGGCATAAGT  -3
A11      TATGTTTTGGTAGTAGCG-----ATGGGGCATAAGT  -5
H11      TATGTTTTGGTAGTAGC------ATGGGGCATAAGT  -6
C11      TATGTTTTGGTAGTAG----------GGGCATAAGT -10
```

```
PDS-S1(M)                                     NcoI   PAM
NbPDS    TATGTTTTGGTAGTAGCGACTCC-ATGGGGCATAAGT 3/5
B05      TATGTTTTGGTAGTAGCGACTCCCATGGGGCATAAGT +1
A05      TATGTTTTGGTAGTAGCG------ATGGGGCATAAGT -5
E05      TATGTTTTGGTAGTAG--------ATGGGGCATAAGT -7

PDS-S2(M)
NbPDS    TATGTTTTGGTAGTAGCGACTCC-ATGGGGCATAAGT 4/5
E06      TATGTTTTGGTAGTAGCGACTCCAATGGGGCATAAGT +1
D06      TATGTTTTGGTAGTAGC-------ATGGGGCATAAGT -6
A06      TATGTTTTGGTAGTAGCGAC----------GGCATAAGT -7
B06      TATGTTTTGG--------------ATGGGGCATAAGT -13

PDS-S3(M)
NbPDS    TATGTTTTGGTAGTAGCGACTCCATGGGGCATAAGT  3/5
A07      TATGTTTTGGTAGTAGCGAC---ATGGGGCATAAGT  -3(X2)
B07      TATGTTTTGGTAGTAGC------ATGGGGCATAAGT  -6(X2)
E07      TATGTTTTGGTAG----------ATGGGGCATAAGT -10

PDS+HcPro-S1 (M)
NbPDS    TATGTTTTGGTAGTAGCGACTCC-ATGGGGCATAAGT 5/5
E08      TATGTTTTGGTAGTAGCGACTCCCATGGGGCATAAGT +1
C08      TATGTTTTGGTAGTAGCGACT-C-ATGGGGCATAAGT -1
A08      TATGTTTTGGTAGTAGCGA-----ATGGGGCATAAGT -4
D08      TATGTTTTGGTAGTAGC-------ATGGGGCATAAGT -6
B08      TATGTTTTGG--------------ATGGGGCATAAGT -13

PDS+HcPro-S2 (M)
NbPDS    TATGTTTTGGTAGTAGCGACTCCATGGGGCATAAGT  2/5
C09      TATGTTTTGGTAGTAGCGAC---ATGGGGCATAAGT  -3
D09      TATGTTTTGGTAGT----------TGGGGCATAAGT -10

PDS+HcPro-S3 (M)
NbPDS    TATGTTTTGGTAGTAGCGACTCC-ATGGGGCATAAGT 3/5
A10      TATGTTTTGGTAGTAGCGACTCCAATGGGGCATAAGT +1
D10      TATGTTTTGGTAGTAGCGACTC--ATGGGGCATAAGT -1
C10      TATGTTTTGGTAGTAGCGAC----ATGGGGCATAAGT -3

PDS+P19-S2 (M)
NbPDS    TATGTTTTGGTAGTAGCGACTCC-ATGGGGCATAAGT 3/5
E11      TATGTTTTGGTAGTAGCGACTCCCATGGGGCATAAGT +1
D11      TATGTTTTGGTAGTAGCGAC----ATGGGGCATAAGT -3
A11      TATGT-------------------ATGGGGCATAAGT -18

PDS+P19-S3 (M)
NbPDS    TATGTTTTGGTAGTAGCGACTCCATGGGGCATAAGT  4/5
E12      TATGTTTTGGTAGTAGCGAC---ATGGGGCATAAGT  -3
C12      TATGTTTTGGTAGTAGCGA----ATGGGGCATAAGT  -4
A12      TATGTTTTGGTAGTAGCG-----ATGGGGCATAAGT  -5
D12      TATGTTTTGGTAGTAGCGAC-----GGGGCATAAGT  -5

PDS-S1(Top)
NbPDS    TATGTTTTGGTAGTAGCGACTCCATGGGGCATAAGT  3/3
A07      TATGTTTTGGTAGTAGCGACTC-ATGGGGCATAAGT  -1
B07      TATGTTTTGGTAGTAG-------ATGGGGCATAAGT  -7(x2)

PDS-S2(Top)
NbPDS    TATGTTTTGGTAGTAGCGACTCCATGGGGCATAAGT  1/3
G07      TATGTTTTGGTAGTA--------ATGGGGCATAAGT  -8

PDS+HcPro-S3(Top)
NbPDS    TATGTTTTGGTAGTAGCGACTCCATGGGGCATAAGT  1/1
H07      TATGTTTTGGTAGTAGCGACTC-ATGGGGCATAAGT  -1
```

Supplemental Figure 6. FoMV-DC*-gNbPDS induced edits recovered from *Cas9 N. benthamiana* at 14 days post inoculation. Sequence analysis of amplicons that were gel purified and cloned. The *Nco*1 recognition site is indicated by the gray box in the wild type (*NbPDS*) sequence and the cleavage site is indicated by the yellow arrow. The proto-spacer adjacent motif (PAM) is indicated by the blue line, and the guide RNA sequence is indicated by the red text. Blue dashes represent nucleotide deletions, blue letters represent nucleotide insertions, and a red dash indicates a gap in the alignment to the guide RNA sequence due to a nucleotide insertion in one of the amplicon sequences. PDS-I# samples are from the leaves infiltrated with FoMV-DC*-gNbPDS. PDS+HCPro-I# samples are from the leaves co-infiltrated with FoMV-DC*-gNbPDS and 35S::HC-Pro. PDS+P19-I# samples are from leaves co-infiltrated with FoMV-DC*-gNbPDS and 35S::P19. PDS-S1 and PDS+P19-S3 are samples from systemic leaves of plants from which the corresponding infiltrated leaf samples were taken. The ratio to the right of the wild type sequences indicate the number of clones that carried an indel out of the total number of clones sequenced for that plant sample. Negative numbers to the right of a sequence show the number of nucleotides deleted, positive numbers show the number of nucleotides inserted, and (x#) shows the number of times that sequence occurred if more than once.
