# Supplementary material for: Protein expression and gene editing in monocots using foxtail mosaic virus vectors
Source: Plant Direct. 2019 Nov 22;3(11):e00181. doi: 10.1002/pld3.181 (PMC6874699; doi:10.1002/pld3.181)
Supplement: Supplementary file 8 [file PLD3-3-e00181-s008.pdf]

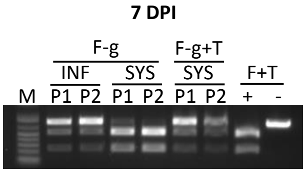

Supplemental Figure 8. TuMV enhances editing in systemic leaves of *Cas9 N. benthamiana* at 7 days post inoculation (DPI) with FoMV-DC*-gNbPDS (F-g). INF, agroinfiltrated leaves; SYS, systemic leaves; M, DNA size marker; P#, plant number; F+T, FoMV-DC* empty vector plus TuMV; +, NcoI added to PCR product; -, not digested PCR product.
