# Supplementary material for: Protein expression and gene editing in monocots using foxtail mosaic virus vectors
Source: Plant Direct. 2019 Nov 22;3(11):e00181. doi: 10.1002/pld3.181 (PMC6874699; doi:10.1002/pld3.181)
Supplement: Supplementary file 9 [file PLD3-3-e00181-s009.pdf]

```
PDS+TuMV-I2                          NcoI   PAM
NbPDS    TATGTTTTGGTAGTAGCGACTCCATGGGGCATAAGT   5/5
G09      TATGTTTTGGTAGTAGCGACTC-ATGGGGCATAAGT   -1(X3)
F09      TATGTTTTGGTAGTAGCGA----ATGGGGCATAAGT   -4
G10      TATGTTTTGGTAGTAG-------ATGGGGCATAAGT   -7

PDS+TuMV-I3
NbPDS    TATGTTTTGGTAGTAGCGACTCCATGGGGCATAAGT   5/5
F12      TATGTTTTGGTAGTAGCGACTC-ATGGGGCATAAGT   -1
F11      TATGTTTTGGTAGTAGCGACT---TGGGGCATAAGT   -3(X2)
G12      TATGTTTTGGTAGTAG-------ATGGGGCATAAGT   -7
G11      TATGTTTTGGTAGT-----------GGGGCATAAGT   -11

PDS+TuMV-I4
NbPDS    TATGTTTTGGTAGTAGCGACTCCATGGGGCATAAGT   5/5
H06      TATGTTTTGGTAGTAGCGACT---TGGGGCATAAGT   -3(X2)
H02      TATGTTTTGGTAGTAGCGAC---ATGGGGCATAAGT   -3
H08      TATGTTTTGGTAGTAGC------ATGGGGCATAAGT   -6(X2)

PDS+TuMV-I5
NbPDS    TATGTTTTGGTAGTAGCGACTCCATGGGGCATAAGT   5/5
G10      TATGTTTTGGTAGTAGCGACT---TGGGGCATAAGT   -3(X2)
F10      TATGTTTTGGTAGTAGCGAC---ATGGGGCATAAGT   -3
F05      TATGTTTTGGTAGTAGC------ATGGGGCATAAGT   -6
G05      TATGTTTTGGTAGTAG-------ATGGGGCATAAGT   -7

PDS+TuMV-S2
NbPDS    TATGTTTTGGTAGTAGCGACTCCATGGGGCATAAGT   3/5
F01      TATGTTTTGGTAGTAGCGACT---TGGGGCATAAGT   -3(X3)

PDS+TuMV-S3
NbPDS    TATGTTTTGGTAGTAGCGACTCCATGGGGCATAAGT   4/5
G04      TATGTTTTGGTAGTAGCGACT---TGGGGCATAAGT   -3
H03      TATGTTTTGGTAGTAGC------ATGGGGCATAAGT   -6(X2)
G03      TATGTTTTGGTAGTAG-------ATGGGGCATAAGT   -7

PDS+TuMV-S4
NbPDS    TATGTTTTGGTAGTAGCGACTCC-ATGGGGCATAAGT  2/5
G05      TATGTTTTGGTAGTAGCGACTCCAATGGGGCATAAGT  +1
F06      TATGTTTTGGTAGTAGCGAC----ATGGGGCATAAGT  -3

PDS+TuMV-S5
NbPDS    TATGTTTTGGTAGTAGCGACTCCATGGGGCATAAGT   2/5
F07      TATGTTTTGGTAGTAGCGACT---TGGGGCATAAGT   -3
H07      TATGTTTTGGTAGTAGC------ATGGGGCATAAGT   -6
```

**Supplemental Figure 9.** FoMV-DC*-gNbPDS induced edits recovered from *Cas9 N. benthamiana* at 7 days post co-inoculation with TuMV. Sequence analysis of amplicons that were gel purified and cloned. The *Nco*1 recognition site is indicated by the gray box in the wild type (*NbPDS*) sequence and the cleavage site is indicated by the yellow arrow. The proto-spacer adjacent motif (PAM) is indicated by the blue line, and the guide RNA sequence is indicated by the red text. Blue dashes represent nucleotide deletions, blue letters represent nucleotide insertions, and a red dash indicates a gap in the alignment to the guide RNA sequence due to a nucleotide insertion in one of the amplicon sequences. PDS-TuMV-I# samples are from the leaves co-infiltrated with FoMV-DC*-gNbPDS and TuMV. PDS-TuMV-S# samples are from the systemic leaves the plants co-infiltrated with FoMV-DC*-gNbPDS and TuMV. The ratio to the right of the wild type sequences indicate the number of clones that carried an indel out of the total number of clones sequenced for that plant sample. Negative numbers to the right of a sequence show the number of nucleotides deleted, positive numbers show the number of nucleotides inserted, and (x#) shows the number of times that sequence occurred if more than once.
