# Supplementary material for: Protein expression and gene editing in monocots using foxtail mosaic virus vectors
Source: Plant Direct. 2019 Nov 22;3(11):e00181. doi: 10.1002/pld3.181 (PMC6874699; doi:10.1002/pld3.181)
Supplement: Supplementary file 10 [file PLD3-3-e00181-s010.pdf]

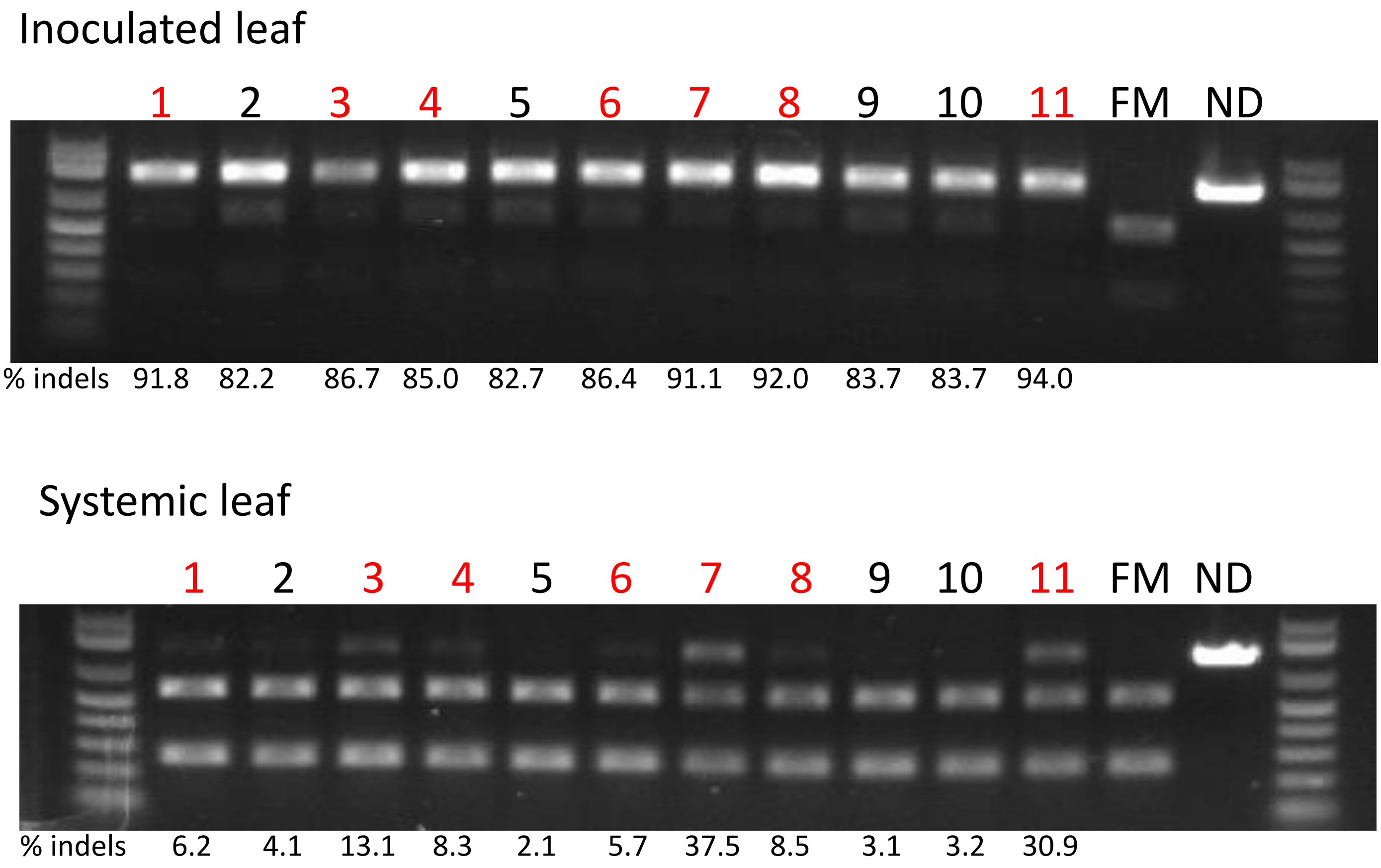

Supplemental Figure 10. *NbPDS* editing in *N. benthamiana* F1 plants from a HcPro x Cas9 cross. The plants indicated in red carried both Cas9 and HcPro, and the plants indicated in black carried only Cas9. The upper panel is from the agroinoculated leaves and the lower panel is from the top systemic leaves. Oligonucleotide primers were used to generate 797 bp amplicons flanking the Pds guide RNA target site, and the amplicons were incubated with *Nco*I. The wild type amplicons are cleaved into two bands of 541 bp and 256 bp, amplicons carrying edits that disrupt the NcoI site are not cleaved. FM was infected with the FoMV empty vector, and ND is the non-digested PCR amplicon. % Indels, percent of each PCR amplicon that was not digested.
