# Supplementary material for: Protein expression and gene editing in monocots using foxtail mosaic virus vectors
Source: Plant Direct. 2019 Nov 22;3(11):e00181. doi: 10.1002/pld3.181 (PMC6874699; doi:10.1002/pld3.181)
Supplement: Supplementary file 11 [file PLD3-3-e00181-s011.pdf]

A

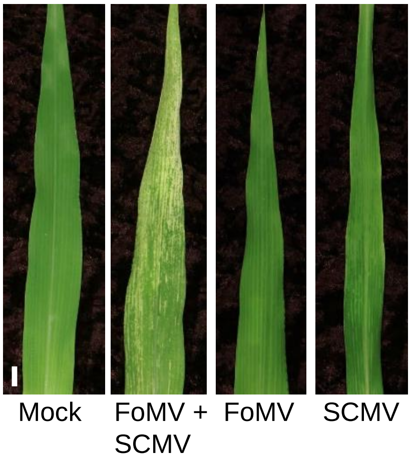

B

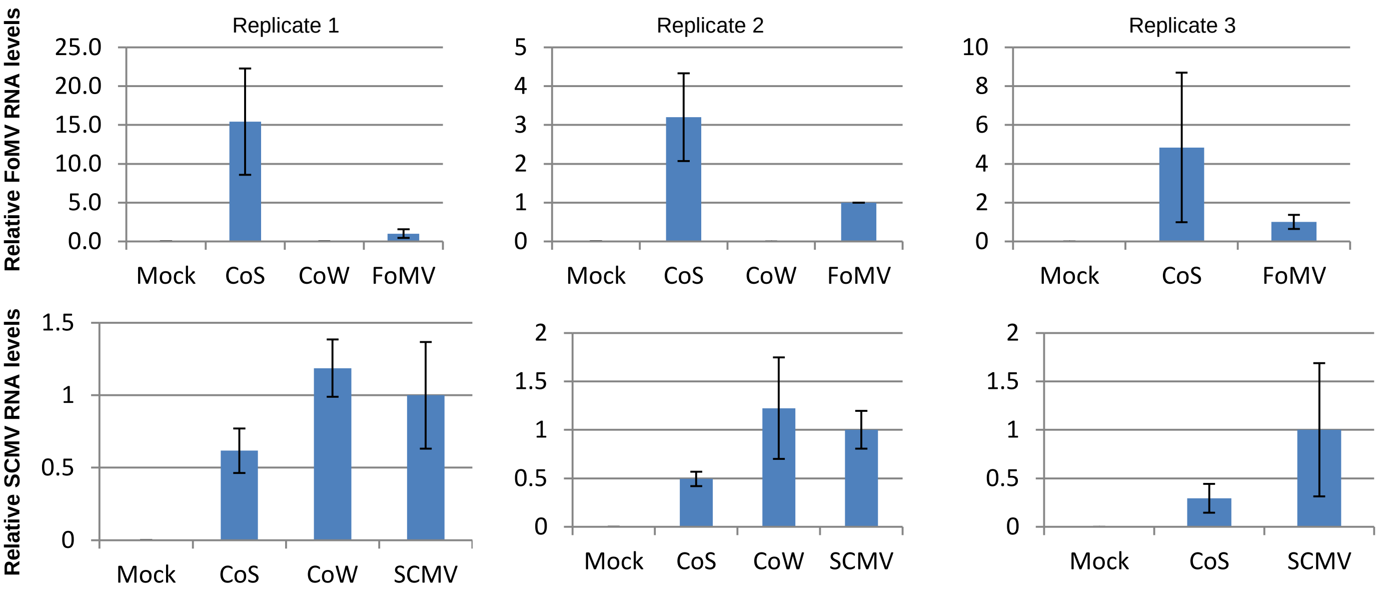

C

	Number of plants in each virus treatment				
Replicate	Mock	CoS	CoW	FoMV	SCMV
Replicate1	3	3	2	3	3
Replicate2	3	3	3	1	3
Replicate3	3	3	NA	5	2

Supplemental Figure 11. Synergism in FoMV + SCMV co-infections. **A.** More severe symptoms are caused by the FoMV + SCMV co-infection as compared to either virus alone. Mock, mock-inoculated plants. **B.** Accumulation of FoMV and SCMV RNAs in single and mixed infections. Bars represent standard deviation (see C for numbers of plants). Mock, mock inoculated, CoS, co-inoculated with FoMV + SCMV and severe symptoms; CoW, co-inoculated with FoMV + SCMV and weak symptoms similar to SCMV alone. The co-inoculation was conducted three independent times and QRT-PCR results for FoMV and SCMV are shown for each replicate. FoMV accumulation was increased in all three replicates but the increase was highly variable. CoW plants were all negative for FoMV PCR product demonstrating that these were only infected with SCMV. SCMV levels did not increase in the co-infections and were typically lower than in the SCMV single infection. **C.** Table summarizes numbers of plants for each treatment in each independent replicate.
