# Supplementary material for: Protein expression and gene editing in monocots using foxtail mosaic virus vectors
Source: Plant Direct. 2019 Nov 22;3(11):e00181. doi: 10.1002/pld3.181 (PMC6874699; doi:10.1002/pld3.181)
Supplement: Supplementary file 12 [file PLD3-3-e00181-s012.pdf]

Supplemental Table 1. Primers and oligos used in this study.

Primer name	Primer sequence
5AmuS1	CGCCGACAACCTACAATACA
5AmuA1	GATTTCGACGTGGCACGATG
5AmuS2	CATCGTGCCACGTCGAAATC
5AmuA2	CGTGAAAATCGTGGAGTCG
201DPA1	GTTGTTAACTCTCCTAAGGACACGTGGAGTGCCAGCAGTTTC
201DPS1	AGGAGAGTTAACAACGGGCCCCACTCAACGACCGCATTG
DPS	5'PhosTCAACGACCGCATTGAGGGTGTTAGGGTAACCAACATCAGTGAAGA GAAACCCTTAGGCACTTT
DPA	AAAGTGCCTAAGGGTTTCTCTTCACTGATGTTGGTTACCCTAACACCCTCA ATGCGGTCGTTGA
DCS	5'PhosACAGCGTCCCCACTGAGGGTGTTAGGGTAACCAACATCAGTGAAGA GAAACCCTTAGGCACTTT
DCA	AAAGTGCCTAAGGGTTTCTCTTCACTGATGTTGGTTACCCTAACACCCTCA GTGGGGACGCTGT
GFP Bsu36I	CCTTAGGATGGTGAGCAAGGGAGAGGA
GFP PspOMI	GGGCCCTCACTTGTACAGCTCGTCCATGC
BAR Bsu36I	CCTTAGGATGAGCCCAGAACGACG
BAR HpaI	GTTAACTCAGATCTCGGTGACGGGCA
Sish1OE1	TTAGGATGTTCAGCATGCTATTACTGTTTTAGAGCTAGAAATAGCAAGTTA AAATAAGGCTAGTCCGTTATCAACTTGAAAAAGTGGCACCGAGTCGGTGC G
Sish1OE2	GGCCCGCACCGACTCGGTGCCACTTTTTCAAGTTGATAACGGACTAGCCTT ATTTTAACTTGCTATTTCTAGCTCTAAAACAGTAATAGCATGCTGAACATC C
Sish1VIGS1	CTAGAATGTTCAGCATGCTATTACTGTTTTAGAGCTAGAAATAGCAAGTTA AAATAAGGCTAGTCCGTTATCAACTTGAAAAAGTGGCACCGAGTCGGTGC C
Sish1VIGS2	TCGAGGCACCGACTCGGTGCCACTTTTTCAAGTTGATAACGGACTAGCCTT ATTTTAACTTGCTATTTCTAGCTCTAAAACAGTAATAGCATGCTGAACATT
CA2OE1	TTAGGTCGACGGTCGTGCAGCTGGAGTTTTAGAGCTAGAAATAGCAAGTTA AAATAAGGCTAGTCCGTTATCAACTTGAAAAAGTGGCACCGAGTCGGTGC G
CA2OE2	GGCCCGCACCGACTCGGTGCCACTTTTTCAAGTTGATAACGGACTAGCCTT ATTTTAACTTGCTATTTCTAGCTCTAAAACTCCAGCTGCACGACCGTCGACC
CA2VIGS1	CTAGATCGACGGTCGTGCAGCTGGAGTTTTAGAGCTAGAAATAGCAAGTT AAAATAAGGCTAGTCCGTTATCAACTTGAAAAAGTGGCACCGAGTCGGTG CC
CA2VIGS2	TCGAGGCACCGACTCGGTGCCACTTTTTCAAGTTGATAACGGACTAGCCTT ATTTTAACTTGCTATTTCTAGCTCTAAAACTCCAGCTGCACGACCGTCGAT
FM-PacIFor	GCTCTAGACCCTTAATTAATGATCAGTAGTATGATACCAATAA
NosRev	AGACCGGCAACAGGATTCA
1380F	AACGCTAGCCACCACCAC
1380R	CAACATGGTGGAGCACGA
DCPacI1380F	GAGAGTGTCGTGCTCCACCATGTTGCATAAGTGCGGCGACGATAG
DCPacI1380R	CGTGGTGGTGGTGGTGGTGGCTAGCGTTGAGGCCCTTTCGTCTTCAAG
ZT-IDF0	AAGGACGGCCATGGCGATTTCGCAT
ZT-IDR0	CCGATGATCTTCTGATAGATGGATC
ZmActS	CCTGAAGATCACCCTGTGCT
ZmActR	GCAGTCTCCAGCTCCTGTTC

ZmHKT1g1	TTAGGGGCTTCGTGCCAACCAACGAGTTTTAGAGCTAGAAATAGCAAGTTAAAATAAGGCTAGTCCGTTATCAACTTGAAAAAGTGGCACCGAGTCGGTGCTTTTTTTG
ZmHKT1g2	GGCCCAAAAAAAGCACCGACTCGGTGCCACTTTTTCAAGTTGATAACGGACTAGCCTTATTTTAACTTGCTATTTCTAGCTCTAAAACTCGTTGGTTGGCACGAAGCCCC
Cas9F?	GGGTAATGAACTCGCTCTGC
Cas9R?	TGGCGTCAAGAACTTCCTTTG
NbPDSs	GAAACACATCACCTAGGCGG
NbPDSa	GGGCGTGAGGAAGTACGAAA
oEK094	GGCATGCACGAATCACACCT
oEK096	CAACCACGGTCAAACAAGGC
oEE374	GAAGGCTTGGACCTTTGGGAG
oEE375	CCACCGCACAAAGGTCAATC
SvCA2g1	CAGTGAAGAGAAACCCTTAGGTCGACGGTCGTGCAGCTGGAGTTTTAGAGCTAGAAATAG
sgRNA:7xT:PspOMI	GTCGTTGAGTGGGGCCCAAAAAAAGCACCGACTCGGTGCCACTT
