# Supplementary material for: Protein expression and gene editing in monocots using foxtail mosaic virus vectors
Source: Plant Direct. 2019 Nov 22;3(11):e00181. doi: 10.1002/pld3.181 (PMC6874699; doi:10.1002/pld3.181)
Supplement: Supplementary file 7 [file PLD3-3-e00181-s007.pdf]

A

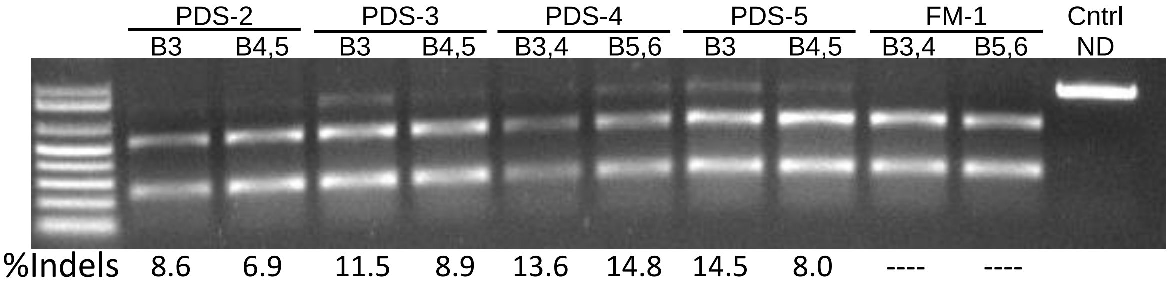

B

```
PDS-2 B3 (sample #1)              NcoI   PAM
NbPDS    TATGTTTTGGTAGTAGCGACTCCATGGGGCATAAGT  2/6
E01      TATGTTTTGGTAGTAGCGACTC-ATGGGGCATAAGT  -1
C01      TATGTTTTGGTAGTAG-------ATGGGGCATAAGT  -7

PDS-2 B4&5 (sample #2)
NbPDS    TATGTTTTGGTAGTAGCGACTCC-ATGGGGCATAAGT  2/8
F02      TATGTTTTGGTAGTA---------ATGGGGCATAAGT  -8
G02      TATGTTTTGGTAGTAGCGACTCCAATGGGGCATAAGT  +1

PDS-3 B3 (sample #3)
NbPDS    TATGTTTTGGTAGTAGCGACTCCATGGGGCATAAGT  4/7
C03      TATGTTTTGGTAGTAGCGA----ATGGGGCATAAGT  -4
F03      TATGTTTTGGTAGTAGC------ATGGGGCATAAGT  -6(X2)
B03      TATGTTTTGGTAGTA--------ATGGGGCATAAGT  -8

PDS-3 B4&5 (sample #4)
NbPDS    TATGTTTTGGTAGTAGCGACTCCATGGGGCATAAGT  3/6
B04      TATGTTTTGGTAGTAGCGAC---ATGGGGCATAAGT  -3(X2)
E04      TATGTTTTGGTAGTAG-------ATGGGGCATAAGT  -7

PDS-4 B3&4 (sample #5)            NcoI   PAM
NbPDS    TATGTTTTGGTAGTAGCGACTCCATGGGGCATAAGT  6/8
B05      TATGTTTTGGTAGTAGCGACTC-ATGGGGCATAAGT  -1(X3)
H05      TATGTTTTGGTAGTAGCGAC---ATGGGGCATAAGT  -3
G05      TATGTTTTGGTAGTAGCG------TGGGGCATAAGT  -6
A05      TATGTTTTGGTAGTAG----------GGGCATAAGT -10

PDS-4 B5&6 (sample #6)
NbPDS    TATGTTTTGGTAGTAGCGACTCCATGGGGCATAAGT  1/5
A06      TATGTTTTGGTAGTAGCGACTC-ATGGGGCATAAGT  -1

PDS-5 B3 (sample #7)
NbPDS    TATGTTTTGGTAGTAGCGACTCCATGGGGCATAAGT  4/7
F07      TATGTTTTGGTAGTAGCGACTC-ATGGGGCATAAGT  -1(X2)
B07      TATGTTTTGGTAGTAG-------ATGGGGCATAAGT  -7
E07      TATGTTTTGGTA-----------ATGGGGCATAAGT -11

PDS-5 B4&5 (sample #8)
NbPDS    TATGTTTTGGTAGTAGCGACTCCATGGGGCATAAGT  3/6
A08      TATGTTTTGGTAGTAGCGACTC-ATGGGGCATAAGT  -1
D08      TATGTTTTGGTAGTAG-------ATGGGGCATAAGT  -7
F08      TATGTTTTGGTAGTAG----------GGGCATAAGT -10
```

Supplemental Figure 7. FoMV-DC*-gNbPDS induced edits recovered from *Cas9 N. benthamiana* flowers approximately 2 months after inoculation. **A.** PCR-based assay to detect edits in *NbPDS*. Oligonucleotide primers were used to generate 797 bp amplicons flanking the *NbPDS* guide RNA target site, and the amplicons were incubated with *Nco*I. The wild type amplicons are cleaved into two bands of 541 bp and 256 bp, amplicons carrying edits that disrupt the *Nco*I site are not cleaved. PDS-# indicates the independent plant that was sampled, B# or B#,# indicates the flower or two flowers that were collected for the DNA extraction, FM-1 is a Cas9 plant infected with FoMV-DC* empty vector, and Cntrl ND is non-digested DNA from the FM-1 plant. **B.** Sequence analysis of amplicons that were gel purified and cloned. The *Nco*1 recognition site is indicated by the gray box in the wild type (*NbPDS*) sequence and the cleavage site is indicated by the yellow arrow. The proto-spacer adjacent motif (PAM) is indicated by the blue line, and the guide RNA sequence is indicated by the red text. Blue dashes represent nucleotide deletions, blue letters represent nucleotide insertions, and a red dash indicates a gap in the alignment to the guide RNA sequence due to a nucleotide insertion in one of the amplicon sequences. The PDS-# and B# correspond to the samples shown in the agarose gel in panel A. The ratio to the right of the wild type sequences indicate the number of clones that carried an indel out of the total number of clones sequenced for that plant sample. Negative numbers to the right of a sequence show the number of nucleotides deleted, positive numbers show the number of nucleotides inserted, and (x#) shows the number of times that sequence occurred if more than once.
