# Supplementary material for: Protein expression and gene editing in monocots using foxtail mosaic virus vectors
Source: Plant Direct. 2019 Nov 22;3(11):e00181. doi: 10.1002/pld3.181 (PMC6874699; doi:10.1002/pld3.181)
Supplement: Supplementary file 13 [file PLD3-3-e00181-s013.pdf]

Reviewer comments:
Reviewer #1 (Comments for the Author):

In the manuscript entitled "Protein expression and gene editing in monocots using foxtail mosaic virus vectors", the authors developed recombinant foxtail mosaic virus clones to express proteins or functional guide RNAs. The most exciting aspect of this work is demonstration that FoMV can deliver functional gRNA in monocot plants. The authors proved that the engineered FoMV vectors can mediate gene editing in Nicotiana benthamiana, Setaria viridis, and Zea mays.

1. In general, the data are rich and well organized, but the novelty and significance are a bit lacking, considering that the engineered FoMV has been reported for VIGS and VOX, moreover, other viruses like TRV has been reported for mediating genome editing. Therefore, the work just expands the utility of FoMV for VIGS, VOX and VEdGE (virus-enabled gene editing) as the authors claimed. More importantly, in my opinion, not inheritable makes this approach less important thus far.

RESPONSE: We thank the reviewer for acknowledging the amount of work and the quality of the work presented in this manuscript. We understand the concern about significance. Unfortunately, another group published on the protein expression aspects of FoMV before we could complete our story that also expands the usefulness of FoMV for delivering guide RNAs in monocot plants. Our story adds detail on FoMV transcription start sites, approaches for stabilizing gene expression, and insert stability that were not previously published by others, so there is some new information in our manuscript concerning gene expression from FoMV. Our study is by far the most thorough analysis virus-mediated guide RNA delivery in monocot species, and we investigate ways in which editing may be enhanced through the use of silencing suppressors and synergistic viruses, which we have not seen in other publications.

2. In L286-294, NbPDS gene editing was detected in the flower tissues of FoMV-DC*-gNbPDS-infected Cas9-overexpression plants, however, the progeny of plants didn't show any photobleached phenotype, implying that genomic modification in the PDS target is not inherited to the next generation. The authors should provide some reasonable explanations here or in the discussion. And if the authors checked the gene editing inheritance in S. viridis as in N. benthamiana.

RESPONSE: FoMV is generally not a seed-transmissible virus, and so the editing detected in the flower was likely occurring only in maternal tissues (i.e. petals and sepals). The editing was also not observed in S. viridis. We have added a sentence on this point at line 337-339. We also added two sentences at lines 465-468 on the reason why editing was not observed in progeny of N. benthamiana and S. viridis with a citation in which it was shown the FoMV is not seed transmitted in S. viridis.

3. In addition, in Char et al., 2017, utilizing Agrobacterium delivered CRISPR/Cas9 for high-frequency targeted mutagenesis has already been applied in maize. However, viral vectors often caused severe symptoms to the plants especially when co-infection with synergistic virus in spite of enhanced efficiency of editing, making it difficult for obtaining the seeds for T1 plants. All of these make this approach inconvenient for studying the heritability of the gene editing. Maybe the authors should

provide more evidences to illustrate the significance of this approach.

RESPONSE: We agree that synergistic viruses are problematic if the goal is to create heritable edits, but that is not the focus of this manuscript. We are demonstrating the feasibility of using FoMV to deliver gRNA. We think the major advantage of this system is that we can use it to rapidly test the performance of gRNAs prior to transformation, which will be very helpful. Also, our goal was to illustrate the potential for using silencing suppressors and other viruses to enhance gene editing. We believe this will be important, as it has for gene expression, but more work is needed to harness/optimize the approach. We have a sentence on this point at line 451-453, which was not changed.

Other major and minor questions are listed below:

4. L300, "the localized expression of the silencing suppressor did not enhance editing", L315, "TuMV HcPro silencing suppressor promotes gene editing", the contradictory conclusions are stated. I speculate that it may result from the difference between transient expression and transgenic overexpression. I think the differences and the possible reasons should be provided and the conclusions should be given more precisely under specific conditions. I suggest that the authors should check the accumulation of guide RNAs.

RESPONSE: The localized versus systemic expression of silencing suppressors is discussed now in lines 424-427. Regarding accumulation of gRNA, we more specifically added this point in the discussion at line 435. This is a current topic of research in our lab, and we intend to publish a more complete story on it after we find strategies to manipulate the synergism so that it will be useful for virus-enabled gene editing.

5. In figure 8A, from the figure legends, it seems that the authors using FoMV-DC-gSvCA2 construct infiltrated into N. benthamiana and rub inoculated onto young S.viridis leaves, not infecting S. viridis directly. But in the context L321-326, this was not clearly elucidated, making the readers confused here.

RESPONSE: Lines 321 – 326 (see lines 325-326) were modified to make it clear that we first agroinoculated N. benthamiana to create inoculum that was rub-inoculated onto S. viridis.

6. L332, Why the frequency of indels are higher in the systemic leaf than in the inoculated leaf?

RESPONSE: The frequency is likely higher here because there is more viral infection in the systemic leaves than in the inoculated leaves. This is not agroinoculation like in N. benthamiana. Therefore, we expect fewer cells to be infected in the inoculated leaves than in the systemic leaves. However, this was not directly tested. We did not modify the text based on this comment.

7. L457, the authors claimed this approach is a powerful application in rapidly screen single gRNA candidates prior to producing gRNA-Cas9 transgenic lines carrying heritable edits. But no such gRNA comparisons are exemplified in the manuscript.

RESPONSE: We have tempered the language of this sentence, which is now at lines 468-473, since it was not directly shown.

8. Figure 8C, why using FoMV-DP-gSvCA2 to test stability of gRNA in sweet corn? The figure 8C seems lacking of positive control.

RESPONSE: We agree that this is confusing and does not add a major point to the results, so we have deleted Figure 8C, the accompanying sentence in the results (lines 342-344), and in the figure legend (lines 731-735).

Reviewer #2 (Comments for the Author):

In this study, the authors used FoMV to build up a transient expression system and guide RNA delivery for Cas9-mediated gene editing in maize, Setaria viridis, and Nicotiana benthamiana. Further, they used RNAi suppressor or co-infection to promote the activity for gene editing. However, there are some shortcomings in their system.

1. FoMV-based expression is very useful for the gene functional study in monocot plants. Unfortunately, a similar FoMV-based transient expression system has been published (Bouton et al., 2018). This hurts the novelty of this work.

RESPONSE: See response to #1 from reviewer 1.

2. FoMV-mediated guide RNA delivery for Cas9-mediated gene editing is just a transient system and cannot be used for obtaining the stable transgenic plants for further study or crop improvement. Further, Co-infection or gene silencing suppressor used in this study will make this system more complicated and useless.

RESPONSE: We think our data point to some interesting future work on using FoMV for gene editing in monocots and for manipulating silencing suppressor or synergistic viruses for enhancing editing and possibly enabling heritable editing, but that will require more experimentation. See response to point #3 from reviewer 1.
